# Bacterial DNAemia is associated with serum zonulin levels in older subjects

**DOI:** 10.1038/s41598-021-90476-0

**Published:** 2021-05-26

**Authors:** Giorgio Gargari, Giacomo Mantegazza, Valentina Taverniti, Cristian Del Bo’, Stefano Bernardi, Cristina Andres-Lacueva, Raul González-Domínguez, Paul A. Kroon, Mark S. Winterbone, Antonio Cherubini, Patrizia Riso, Simone Guglielmetti

**Affiliations:** 1 Division of Food Microbiology and Bioprocesses, Department of Food, Environmental and Nutritional Sciences (DeFENS), University of Milan, Via Celoria 2, 20133 Milan, Italy; 2 Division of Human Nutrition, Department of Food, Environmental and Nutritional Sciences (DeFENS), University of Milan, Via Celoria 2, 20133 Milan, Italy; 3 Biomarkers and Nutrimetabolomics Laboratory, Department of Nutrition, Food Sciences and Gastronomy, University of Barcelona, Barcelona, Spain; 4 CIBER de Fragilidad y Envejecimiento Saludable (CIBERFES), Instituto de Salud Carlos III, Barcelona, Spain; 5 Quadram Institute Bioscience, Norwich Research Park, Norwich, UK; 6 Geriatria, Accettazione geriatrica e Centro di ricerca per l’invecchiamento, IRCCS INRCA, Ancona, Italy

**Keywords:** Gastroenterology, Molecular medicine, Risk factors, Biomarkers, Diagnostic markers, Predictive markers, Prognostic markers

## Abstract

The increased presence of bacteria in blood is a plausible contributing factor in the development and progression of aging-associated diseases. In this context, we performed the quantification and the taxonomic profiling of the bacterial DNA in blood samples collected from forty-three older subjects enrolled in a nursing home. Quantitative PCR targeting the 16S rRNA gene revealed that all samples contained detectable amounts of bacterial DNA with a concentration that varied considerably between subjects. Correlation analyses revealed that the bacterial DNAemia (expressed as concentration of 16S rRNA gene copies in blood) significantly associated with the serum levels of zonulin, a marker of intestinal permeability. This result was confirmed by the analysis of a second set of blood samples collected from the same subjects. 16S rRNA gene profiling revealed that most of the bacterial DNA detected in blood was ascribable to the phylum *Proteobacteria* with a predominance of the genus *Pseudomonas*. Several control samples were also analyzed to assess the influence of contaminant bacterial DNA potentially originating from reagents and materials. The data reported here suggest that para-cellular permeability of epithelial (and, potentially, endothelial) cell layers may play an important role in bacterial migration into the bloodstream. Bacterial DNAemia is likely to impact on several aspects of host physiology and could underpin the development and prognosis of various diseases in older subjects.

## Introduction

The implementation of metagenomics in microbial ecology led to speculate that the dogma of complete sterility of several tissues of the human body, such as amniotic fluid, placenta, bladder, meconium, and blood, could be confuted^1–3^, although the presented data were often controversial^4–8^. In particular, several studies in the last decade reported the existence of bacterial DNA in the bloodstream of healthy individuals^3^. For instance, in 2001, Nikkari et al. reported the presence of significant amounts of bacterial DNA in venous blood specimens from four healthy subjects, and hypothesized the existence of a “normal” population of hematic bacterial DNA molecules^9^. Nonetheless, the precise consequences on host physiology of such bacterial DNAemia are still largely undefined and potentially underestimated^10,11^.

Bacterial DNA in blood plausibly originates from (1) direct translocation of microbial cells through an injured epithelial barrier, (2) microbe sampling activity of antigen-presenting cells (dendritic cells, macrophages) at the epithelium, or (3) via microfold (M) cells in Peyer's patches of the intestine^10^. These events increase the presence of bacterial factors found systemically (in bloodstreams and organs), resulting in a significant stimulation of the host’s immune system. In line with this assumption, blood microbiota abundance and composition were reported to be associated with chronic, inflammatory diseases^10^ and non-communicable diseases such as type II diabetes^12,13^ and cardiovascular disease^14^. Indeed, the presence/size of a blood microbiota population was proposed as a biomarker for the assessment of cardiovascular risk^14^. Overall, the experimental evidence suggests that higher levels of bacterial DNA in blood may be indicative of increased risk of disease, particularly for subjects characterized by other concomitant risk factors, such as older subjects.

Aging is typically characterized by chronic low-grade systemic inflammation (inflammaging;^15^), and it’s severity was reported to be a valid predictor of overall illness and mortality in an older population^16^. Thevaranjan et al. demonstrated in a mouse model that age-related microbial dysbiosis increases intestinal permeability, permitting the translocation into the bloodstream of microbial products that, consequently, trigger systemic inflammation^17^). Therefore, this report provides evidence that supports the notion of a mechanistic link between bacterial DNAemia and inflammaging. In addition, it supports the hypothesis that intestinal barrier deterioration occurs during aging, promoting increased absorption of luminal factors^18^, and supports the now more than 100-year-old Metchnikoff speculation that, during aging, “the intestinal microbes or their poisons may reach the system generally and bring harm to it”^19^.

In this study, we started from the notion that bacterial translocation in blood may play a significant role in the development and progression of aging and aging-associated diseases. To preliminary assess this hypothesis, we performed the quantification and the taxonomic profiling of the bacterial DNA in blood collected from a group of forty-three older subjects; then, we correlated these data with several metabolic and functional markers, including zonulin as marker of intestinal permeability. To the best of our knowledge, this is the first report of the amounts and sources of bacterial DNA in the peripheral blood of a group of older subjects.

## Subjects and methods

### Older population

This study involved the analysis of samples obtained from a group of older volunteers (n = 43; 28 women and 15 men; average age: 79.2 ± 9.8 year; body mass index (BMI): 26.5 ± 5.8; Table 1) enrolled within the project entitled “*Gut and blood microbiomics for studying the effect of a polyphenol-rich dietary pattern on intestinal permeability in the elderly*” (MaPLE project)*.* Subjects were recruited in a well-controlled setting at Civitas Vitae (OIC Foundation, Padua, Italy that include residential care and independent residences for older subjects). In particular, older subjects were specifically excluded from the study if taking regularly medications known to have a direct effect on intestinal permeability (e.g. aspirin, buprofen, antibiotics, chemotherapy drugs). Specific inclusion and exclusion criteria were previously reported in detail^20^.

**Table 1:** Demographic and anthropometrical characteristics of older volunteers recruited for the study.

Subject (n = 43)	Sex (28F/15 M)	Age (62–98 yo)	BMI (kg/m^2^)	Bacterial load (16S rRNA g.c./μl)		Zonulin level (ng/ml)	
				*I*	*II*	*I*	*II*
OS01	M	75	20.5	1539	2296	50.1	36.5
OS02	F	81	22.0	4694	3992	48.9	43.9
OS03	F	75	22.1	4480	2050	36.8	30.6
OS04	F	80	26.7	8959	8213	54.7	55.2
OS05	F	74	30.3	2075	5765	35.8	31.6
OS06	F	88	33.4	2267	6676	52.2	56.0
OS07	M	93	24.5	3752	3087	25.7	37.0
OS08	F	88	30.7	5023	5319	42.3	39.2
OS09	M	72	21.0	7137	3393	47.2	28.0
OS10	F	85	29.9	6203	10,166	36.4	59.9
OS11	M	72	23.5	3277	3437	35.5	30.0
OS12	M	82	22.7	1777	6592	32.8	30.2
OS13	F	71	19.6	1455	3601	48.1	26.9
OS14	M	71	18.1	1715	2554	20.6	28.6
OS15	F	95	29.7	10,491	12,955	61.6	40.2
OS16	M	85	29.2	5812	5169	36.2	35.8
OS17	F	91	23.8	5031	4713	22.2	30.6
OS18	F	86	25.4	7428	3529	29.5	45.0
OS19	F	88	20.4	8423	6241	40.0	42.9
OS20	M	86	30.8	3538	1599	27.5	32.7
OS21	M	95	26.4	11,716	*n.a*	55.4	*n.a*
OS22	F	98	25.2	3507	3271	25.4	33.5
OS23	F	81	33.2	3576	4337	34.5	38.2
OS24	F	76	30.8	7305	7406	49.4	57.7
OS25	F	67	18.4	5781	4803	45.7	40.5
OS26	F	66	22.8	5812	5813	43.4	46.3
OS27	F	65	21.1	1999	2197	38.2	38.2
OS28	M	91	28.0	3944	4175	44.6	39.7
OS29	F	73	30.5	12,405	12,782	46.9	47.2
OS30	F	90	21.3	6509	2507	39.9	53.4
OS31	M	80	46.1	3415	5171	34.3	35.7
OS32	F	76	22.5	1271	4014	26.9	27.2
OS33	F	87	31.2	7137	3976	30.7	37.8
OS34	F	90	31.9	8193	6682	49.2	45.6
OS35	F	79	33.9	12,482	3619	75.8	50.8
OS36	M	67	28.7	9955	10,265	51.6	33.5
OS37	M	63	20.7	11,410	4340	49.4	42.2
OS38	F	79	25.7	4250	4656	48.3	39.5
OS39	F	77	19.0	5356	2069	39.4	36.0
OS40	M	64	24.3	6547	2694	37.8	31.1
OS41	F	62	38.3	5444	3952	30.5	31.2
OS42	F	73	24.8	7382	4348	42.5	35.2
OS43	M	69	30.1	7887	4461	45.0	53.6

Blood bacterial load (determined by qPCR and expressed as 16S rRNA gene copies per μl) and zonulin level (determined by ELISA test) are also shown. OS01-43, consecutive anonymized coding of the older subjects included in the study. 16S rRNA g.c., 16S rRNA gene copies; *I* and *II*, first and second set of blood samples analyzed, respectively; *n.a.*, data not available due to missing sample.

### Blood sampling

Blood drawing from a peripheral vein was performed early in the morning from fasted subjects. In detail, skin was cleaned with 100% hydrophilic cotton wool soaked in denatured ethyl alcohol, immediately before the introduction into vein of the needle of a vacutainer connected with a 5 ml EDTA tube (all material was from Becton Dickinson Italia S.p.A., Milan, Italy). Immediately after collection, blood was transferred into 2 ml certified DNase/RNase free and pyrogen free cryovials (Sigma-Aldrich S.r.l., Milano, Italy) using PCR-grade filtered pipettes tips (Corning® Isotip® filtered pipet tips), in order to prepare two 0.5 ml aliquots that were immediately stored at − 80 °C until DNA extraction. The same material was used for control samples. Remaining blood was centrifuged at 1000 × g for 15 min at room temperature and the obtained serum stored at − 80 °C for the subsequent analysis of metabolic/functional markers.

### DNA extraction, qPCR experiments and sequencing of 16S rRNA gene amplicons

Bacterial DNA quantification and sequencing reactions were performed by Vaiomer SAS (Labège, France) using optimized blood-specific techniques as described earlier^3,21^. All blood and control samples of this study are reported in Table 2.

**Table 2:** List of samples analyzed in the study and simultaneity of the experimental steps.

Sample	*n*	Experiment simultaneity		
		DNA extraction	qPCR	16S rRNA gene profiling
Blood	43	A	A	A
C_extr_	4	B	B	*n.p.*
C_dEDTA_	1			
C_vEDTA_	1			
C_qPCR_	4			B
Blood	42	C	C	C
Blood	1	D	D	D
C_dEDTA_	1			
C_vEDTA_	1			
C_extr_	4			

Samples with the same letter (A, B, C or D) have been concurrently processed. C_extr_, ultrapure water; C_dEDTA_, commercial phosphate buffered saline (PBS) added directly into an EDTA tube; C_vEDTA_, PBS added into an EDTA tube after passage through a vacutainer blood collection system; *n*, number of samples; *n.p.*, analysis not performed for the respective samples.

Since low microbial biomass was expected, four technical controls, consisting of ultrapure water (UPW), were analyzed in the same way as the blood samples (C_extr_). Two additional control samples (C_EDTA_) were prepared by adding 2 ml of commercial phosphate buffered saline (PBS; Sigma-Aldrich) into an EDTA tube, directly (C_dEDTA_) or after passage through a vacutainer blood collection system (C_vEDTA_). The DNA from these controls was not extracted simultaneously with blood samples. Furthermore, additional four C_extr_, one C_dEDTA_, one C_vEDTA_, and a blood sample collected from one of the volunteers, were simultaneously subjected to DNA extraction, qPCR, and 16S rRNA gene profiling (Table 2). In brief, DNA was extracted from 100 µl of whole blood or control samples and quantified by quantitative real-time PCR (qPCR) experiments using bacterial primers EUBF 5′-TCCTACGGGAGGCAGCAGT-3′ and EUBR 5′-GGACTACCAGGGTATCTAATCCTGTT-3′^22^, which target the V3-V4 hypervariable regions of the bacterial 16S rRNA gene with 100% specificity (i.e., no eukaryotic, mitochondrial, or Archaea DNA is targeted) and high sensitivity (16S rRNA of more than 95% of bacteria in Ribosomal Database Project database are amplified). Four samples of UPW were used as templates in qPCR reactions for technical control (C_qPCR_). The 16S rRNA gene was quantified by qPCR in triplicate and normalized using a plasmid-based standard scale. Finally, the results were reported as number of copies of 16S rRNA gene per µl of blood.

DNA extracted from whole blood and controls (according to Table 2) were also used for 16S rRNA gene taxonomic profiling using MiSeq Illumina technology (2 × 300 paired-end MiSeq kit V3, set to encompass 467-bp amplicon) according to specific protocol and primers from Vaiomer as previously described^3,23,24^. Then, sequences were analyzed using Vaiomer bioinformatic pipeline to determine bacterial community profiles. Briefly, after demultiplexing of the bar-coded Illumina paired reads, single read sequences were trimmed (reads R1 and R2 to respectively 290 and 240 bases) and paired for each sample independently into longer fragments, non-specific amplicons were removed and remaining sequences were clustered into operational taxonomic units (OTUs, here also called “Cluster”) using FROGS v1.4.0^25^ with default parameters. A taxonomic assignment was performed against the Silva 128 Parc database, and diversity analyses and graphical representations were generated using the R PhyloSeq v1.14.0 package. For bacterial taxonomic profiling and correlation analyses, the concentration of each taxon was used both in relative (percentage) abundance and after normalization against the total number 16S rRNA gene copies in that specific sample. The OTU tables including all samples analyzed through 16S rRNA gene profiling in this study are reported in Additional file 2.

### Assessment of the “carrier effect”

The potential influence of nucleic acids from blood to affect the efficiency of extraction of DNA from potential bacterial contaminants was assessed using DNA from λ phage as carrier. In specific, we prepared serial 1:10 dilutions of λ phage DNA (Thermo Fisher Scientific Inc., Monza, Italy) in Milli-Q water (from 2 × 10^6^ to 2 × 10^2^ λ copies per μl). Then, 50 μl of each dilution was added to an EDTA tube (the same tubes used to collect blood) containing 2.7 ml of MRD solution (Scharlab Italia srl, Riozzo di Cerro al Lambro, Italy). A total quantity of λ copies from 10^8^ to 10^4^ were added to EDTA tubes. In addition, carrier effect was assessed adding in the EDTA tubes the DNA extracted from the human leukemic monocyte cell line THP-1 (Sigma-Aldrich); in specific, we added 6 μg of THP-1 DNA to EDTA tubes because this was the amount of total DNA obtained on average from blood samples. Subsequently, DNA was extracted from the 200 μl of each sample (in duplicate) and used in qPCR experiments (in technical duplicate) with the panbacterial primers used for blood samples.

### Blood parameters analyses

Fasting serum levels of glucose, insulin, creatinine, uric acid, aspartate aminotransferase (AST), alanine aminotransferase (ALT), and gamma-glutamyl transferase (GGT), triglyceride (TG), total cholesterol (TC), low-density lipoprotein cholesterol (LDL-C) were determined by an automatic biochemical analyzer (ILAB650, Instrumentation Laboratory and TOSO AIA 900, Italy), while the high-density lipoprotein cholesterol (HDL-C) concentration was estimated using the Friedewald formula^20^. The homeostatic model assessment (HOMA) index was calculated with the formula HOMA-IR = [fasting glucose (mg/dL) × insulin (IU)/405]. Serum zonulin levels were quantified using IDK® Zonulin ELISA Kit (Immundiagnostik AG, Germany). C-reactive protein (CRP) was quantified using the Quantikine Human C-reactive protein ELISA kit (R&D Systems cat# DCRP00), TNF-α using the Quantikine high sensitivity Human TNF-α ELISA kit (R&D Systems cat# HSTA00E) and IL-6 using the Quantikine high sensitivity Human IL-6 ELISA kit (R&D Systems cat# HS600B). Intercellular adhesion molecule-1 (ICAM-1) and vascular cell adhesion molecule-1 (VCAM-1) were quantified in serum samples with an ELISA kit (Booster® from Vinci Biochem S.r.l., Vinci, Italy). The Cockcroft-Gault equation was used for estimating creatinine clearance (CG index).

### Statistics

Statistical analyses were performed using R statistical software (version 3.1.2). The correlation analyses were carried out using the Kendall and Spearman analysis, as previously described^26,27^, calculated between blood bacterial load, age, BMI, and clinical and molecular parameters quantified in the blood of older volunteers at recruitment. The regression analysis was performed to evaluate the linear relationship between total bacteria load and zonulin levels, in addition to Pearson’s correlation analysis. Statistical significance was set at *P* ≤ 0.05; differences with 0.05 < *P* ≤ 0.10 were accepted as trends. When *P* values correction was applied, the Holm-Bonferroni method was used. Normality was assessed by the Shapiro–Wilk test. The following variables passed the normality test (alpha = 0.05): 16S rRNA g.c., zonulin, age, creatinin, total cholesterol, HDL-C, LDL-C, and the LDL/HDL-C ratio.

### Ethics statement

Authors declare that all procedures were in accordance with the ethical standards of the Helsinki Declaration of 1975 as revised in 2013. The study protocol was approved by the Research Ethics Committee of the Università degli Studi di Milano (opinion no. 6/16; February 15th, 2016). All participating subjects provided their written informed consent for the enrolment.

## Results

### Older subjects harbor bacterial DNA in blood

**Figure 1:**
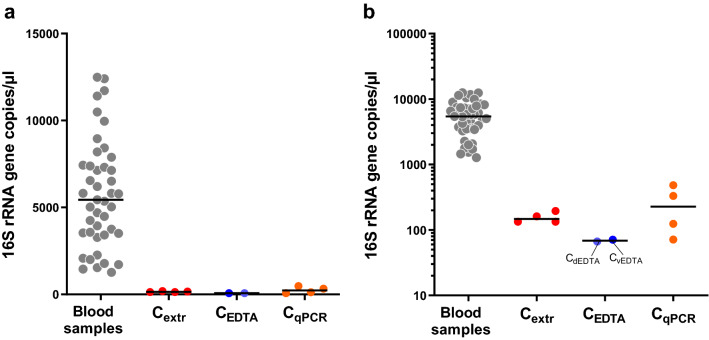
Bacterial load in blood samples of older volunteers (n = 43) and in technical controls as determined by qPCR with panbacterial primers targeting the 16S rRNA gene. Data are reported as copies of the gene per volume of blood in linear (**A**) and logarithmic (**B**) scale. C_extr_, DNA extracted from ultrapure deionized water with the same protocol used for blood samples; C_EDTA_, DNA extracted with the same protocol used for blood samples from phosphate buffered saline (PBS) into an EDTA tube, added directly (C_dEDTA_) or after passage through a vacutainer blood collection system (C_vEDTA_); C_qPCR_, ultrapure deionized water directly used as template in qPCR reactions. Horizontal black lines indicate the median.

qPCR assays with pan-bacterial primers targeting the 16S rRNA gene were used to detect and quantify DNA of bacterial origin in the blood samples collected from 43 older people. In addition, the same analysis was performed on 10 control samples: Extraction controls (C_extr_; four samples), EDTA tube and vacutainer system controls (C_dEDTA_ and C_vEDTA_; two samples) and qPCR controls (C_qPCR_ controls; four samples). Bacterial DNA was detected in all blood samples collected from the older subjects under study. Specifically, we quantified a minimum of 1271 and a maximum of 12,482 copies of the 16S rRNA gene per µl of blood (Table 1 and Fig. 1). Conversely, the gene copies detected in controls were much lower than those found in blood samples, with a mean detection (± standard deviation) of 156 (± 29), 69 (± 3) and 252 (± 190) 16S rRNA gene copies per equivalent μl of blood for C_extr_, C_EDTA_, and C_qPCR_ samples, respectively (Fig. 1).

Overall, these results indicate that the older volunteers under study harbored detectable amounts of bacterial DNA in their blood, in a concentration that varied approximately of one order of magnitude among samples.

### The amount of bacterial DNA in blood of older subjects is associated with serum zonulin

**Figure 2:**
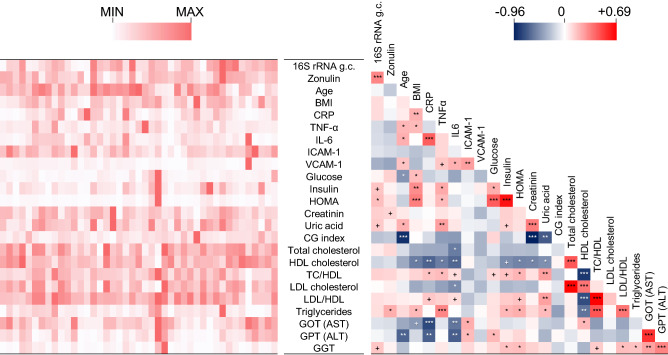
Correlations between bacterial DNAemia, age, BMI, and metabolic and functional markers determined in blood of the older subjects under study (n = 43). The heatmaps on the left (white-red color gradient) refers to the distribution among older subject of the item reported in the central column. The heatmap on the right (blue-white-red color gradient) represents the Spearman’s correlation coefficient, ρ. Asterisks indicate the Kendall rank correlation *P* value: ***P < 0.001; **P < 0.01; *P < 0.05; + , 0.05 < *P* ≤ 0.10.

**Figure 3:**
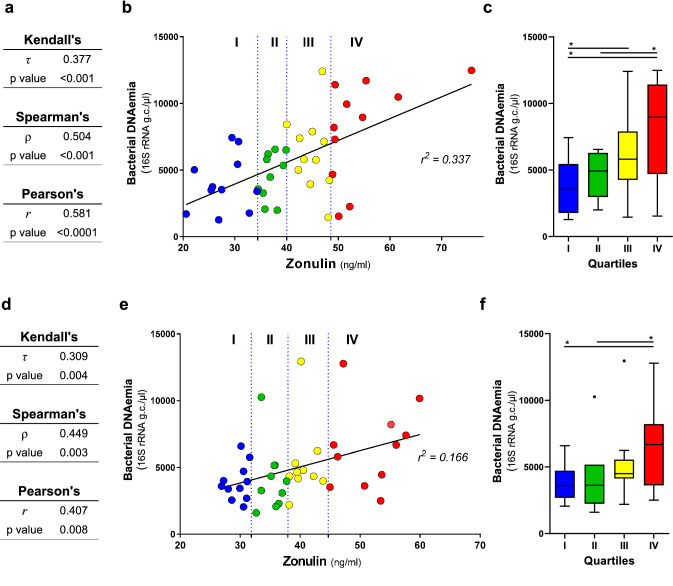
Linear regression and correlation analysis carried out between blood bacterial load and serum zonulin levels. Correlation has been assessed through Pearson’s (*r* coefficient), Spearman’s (ρ), and Kendall’s (τ) analyses. Analyses were performed on the first (n = 43, panels **A**,**B,C**) and the second (n = 42, panel **D**,**E,F**) set of blood samples collected from older people. The second set of blood samples was collected approximately four months after the first draw from the same older volunteers. Panels A and D, tables showing correlation and regression indexes and *p* values. Panels B and E, linear regression plot; vertical dotted lines indicate the quartiles. Panels C and F, Tukey box plots showing levels of bacterial DNAema in blood samples grouped in quartiles based on zonulin level.

In the subsequent part of the study, we performed correlation analyses between the blood bacterial DNA load and several clinical and molecular parameters that are relevant for metabolic and physiological function in the older population (the characteristics of the 43 older subjects involved in this analysis are reported in Table S1). Specifically, besides age and BMI, markers related to immune status (serum CRP, IL-6, and TNF-α concentrations), vascular function (serum ICAM-1 and VCAM-1), glucose metabolism/homeostasis (serum glucose, insulin, and the derived HOMA index), renal function (urinary creatinine, uric acid, and CG index), lipid metabolism (serum TC, HDL-C, LDL-C, and TG), liver function (serum AST, ALT, and GGT) and epithelial permeability (serum zonulin) were also quantified and possible associations with blood bacterial DNA load investigated. A number of expected strong associations were observed, for example between TC and LDL cholesterol, insulin and HOMA index, IL-6 and CRP, and GOT (AST) and GPT (ALT) (Fig. 2). In addition, we found clear inverse correlations of the CG index with creatinine and age, and between GOT (AST) and CRP. We also found a significant correlation between the 16S rRNA gene copy number and HOMA index, and between zonulin and triglycerides. However, in the context of the present study, the most interesting result was the significant positive association between blood bacterial DNA and zonulin (Fig. 2). Specifically, the 16S rRNA gene copy number was found to significantly correlate with serum zonulin levels (Spearman’s ρ = 0.504; Kendall’s *P* = 0.0004; Figs. 2, 3A). We also found a weak but significant linear association (Pearson’s *P* < 0.0001; *r*^2^ = 0.337; Fig. 3B,C).

In order to confirm the observed direct correlation between bacterial DNA load in peripheral blood and serum zonulin levels, blood was collected again from the same cohort of older subjects after approximately four months. Then, 16S rRNA gene copies and zonulin levels were quantified and the results used for correlation and regression analyses. One subject was not included since sample was not available (total samples considered, n = 42). The obtained results revealed again the significant correlation between 16S rRNA gene copies and zonulin levels in blood samples (Spearman’s ρ = 0.4449; Kendall’s *P* = 0.004; Fig. 3D). In addition, also Pearson’s correlation resulted significant (*P* = 0.008), although the linear association was very low (*r*^2^ = 0.166; Fig. 3E,F).

Finally, the comparison of results obtained from blood samples collected before and after 4 months revealed a higher inter-subject than intra-subject variability for zonulin and bacterial DNA abundances. In fact, we found a significant correlation and linear association between the two set of samples for both bacterial DNA load (Spearman’s ρ = 0.379; Kendall’s *P* = 0.013; Pearson’s *P* = 0.010) zonulin levels (Spearman’s ρ = 0.526; Kendall’s *P* < 0.001; Pearson’s *P* = 0.001).

Overall, these results indicate that circulating bacterial DNA is stably linked with serum zonulin level in the group of relatively healthy older people that were studied.

### Taxonomic profiling of blood bacterial DNA

The same blood DNA samples used in qPCR experiments (n = 43) were also used in 16S rRNA gene profiling analysis to define their bacterial taxonomic composition. In addition, in the same analysis, we also taxonomically profiled four C_qPCR_ samples, since they showed the highest concentration of 16S rRNA gene copies among controls (Fig. 1). The average number of reads assigned to OTUs was around 35,000 per sample. Curve plots for rarefaction analysis suggested that the sequence depth captured the diversity in all samples (Fig. S1A). Notably, the OTU richness of blood samples (mean ± standard deviation = 40 ± 8 OTUs per sample) was significantly higher (twice, on average) than the richness of the controls (mean ± standard deviation = 21 ± 2 OTUs per sample) (Fig. S1B). In addition, multidimensional scaling (MDS) representation of β-diversity calculated through generalized UniFrac distance clustered controls outside the group of blood samples (Fig. S1C).

**Figure 4:**
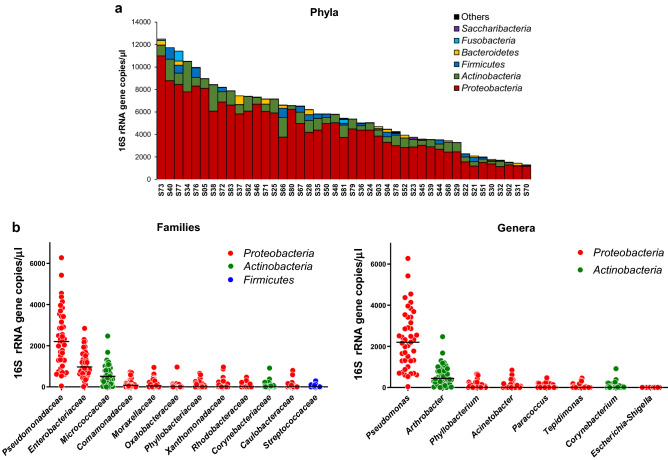
Taxonomic composition of the bacterial DNA detected in the blood of older subjects expressed as 16S rRNA gene copies per volume of blood. (**A**) distribution of phyla in each analyzed blood sample. (**B**) most abundant families and genera detected in the blood samples under study. Horizontal black lines indicate the median.

Subsequently, we analyzed the bacterial composition of blood DNA samples at different taxonomic levels. In order to infer information concerning taxonomic abundance, we normalized the number of reads by multiplying the relative abundance of taxa by the 16S rRNA gene abundance determined by qPCR in each blood sample. This analysis revealed that the phylum *Proteobacteria* was the most represented in all samples, followed by *Actinobacteria*. These two phyla were found in all samples, whereas *Firmicutes* and *Bacteroidetes* were found in 98% and 79% of subjects, respectively (Fig. 4A). *Fusobacteria* were sporadically detected (in about 28% of samples), whereas other phyla were found in less than 5% of samples. Only three families, i.e. *Enterobacteriaceae*, *Pseudomonadaceae* (phylum *Proteobacteria*), and *Micrococcaceae* (phylum *Actinobacteria*) were found in all samples (Fig. 4B). *Pseudomonas* was the most abundant genus in all samples except one, in which the *Proteobacteria* genus *Massilia* was predominant. The second most abundant genus was *Arthrobacter* (family *Micrococcaceae*), followed by the *Proteobacteria* genera *Phyllobacterium* (family *Phyllobacteriaceae*), *Acinetobacter* (family *Moraxellaceae*), *Paracoccus* (family *Rhodobacteraceae*), and *Tepidimonas* (family *Comamonadaceae*) (Fig. 4B). Similar results were found also when the same taxonomic distribution analysis was performed with relative abundance data (i.e., without normalization by the 16S rRNA gene abundance) (Fig. S1D).

Finally, the most abundant OTUs (i.e., having a median abundance > 100 16S rRNA gene copies per μl of blood; clusters 1 to 4) were further taxonomically investigated by manual searching against the 16S rRNA gene database within the GenBank by Basic Local Alignment Search Tool (BLAST). The DNA sequence characterizing Cluster 1 showed 100% similarity with species *Pseudomonas fluorescens* and *Pseudomonas canadensis*. Cluster 2 showed 100% similarity with several *Enterobacteriaceae* species (e.g. *Brennaria alni*, *Escherichia coli*, *Escherichia fergusonii*, *Pseudescherichia vulneris*, *Shigella flexneri*, *Shigella sonnei*). Cluster 3 was 100% similar to *Pseudomonas azotoformans*, *Pseudomonas lactis*, and *Pseudomonas paralactis*, which are species within the *P. fluorescens* species group^28^. Finally, the highest match for Cluster 4 was found with the species *Arthrobacter russicus* (99.8%).

Overall, these results suggest that the bacterial DNA detected in the peripheral blood of older subjects is mostly ascribable to the Gram-negative phylum *Proteobacteria*, with the predominance of the genus *Pseudomonas*.

### Assessment of the role of contaminants on taxonomic profiling data

In order to find potential contaminants deriving from the 16S rRNA gene profiling procedure, the taxonomic composition of the DNA from C_qPCR_ samples, which showed the highest signal among controls in qPCR (Fig. 1), was analyzed and the abundance of bacterial families and genera was compared between blood and controls. After normalization to 16S rRNA gene copies, the abundance of bacterial taxa in control samples was much lower than that of blood samples (Fig. S2A,B). Consequently, we compared the bacterial taxa in blood *vs* control samples without normalization, i.e. using the relative abundance (percentages) (Fig. S2C,D). This comparison revealed comparable relative abundance between controls and blood samples only for families *Enterobacteriaceae* and *Micrococcaceae* and the genera *Arthrobacter* and *Escherichia*/*Shigella*. In addition, *Moraxellaceae* and the genus *Acinetobacter* were higher compared to blood samples for only one of the four control samples. Finally, also *Pseudomonadaceae* and the genus *Pseudomonas* were detected in controls, but in lower percentages than largely most of the blood samples (Fig. S2).

To find potential contaminants deriving from any reagent and material used in the analysis, successively, we also taxonomically profiled by 16S rRNA gene sequencing four C_extr_ control samples, and the two C_EDTA_ controls together with one blood sample collected from an older volunteer. Again, the amount of bacterial DNA detected in control samples was much lower than that of the blood sample (Fig. S3A). About 30 thousand sequencing reads were generated per samples, apart from sample C_dEDTA_, for which approximately 13,000 were obtained (Fig. S3B). Analysis of sequencing reads indicated a wide taxonomic variability between the control samples, whereas the blood samples comprised a bacterial community structure resembling that observed in the older subjects’ blood samples previously analyzed (Fig. S3C,D), which was characterized by *Pseudomonas* as the most abundant genus. The second most abundant bacterial group in the blood sample was ascribed to *Escherichia*/*Shigella*, and it was noted that this taxonomic unit was similarly abundant, in percentage terms, in several control samples. All the other taxonomic units found with a relative abundance higher than 1% in the blood sample were not detected in controls or found in very low concentrations (Fig. S3C,D).

Finally, we also tested the potential contribution of a “carrier effect”, i.e. the ability of additional nucleic acids (such as the host’s DNA and RNA that are abundantly present in blood) to enhance the recovery of DNA from low-abundant microbial cells in the sample^29^. To this aim, serial dilutions of lambda phage DNA were added to EDTA tubes and extracted with the same protocol used for blood samples. The same experiment was also performed using as carrier the DNA isolated from the human cell line THP-1. Then, DNA samples were used in qPCR. The obtained cycle threshold did not change accordingly to the presence of carrier DNA (Fig. S4), suggesting that a “carrier effect” due to human DNA from blood did not occur or was negligible in the experimental settings of our study.

Overall, these data suggest that the contribution of contaminant DNA to the presented taxonomic data cannot be excluded but does not affect significantly most of the dominant taxa reported in blood.

### Serum zonulin is associated to specific bacterial taxa in blood

**Figure 5:**
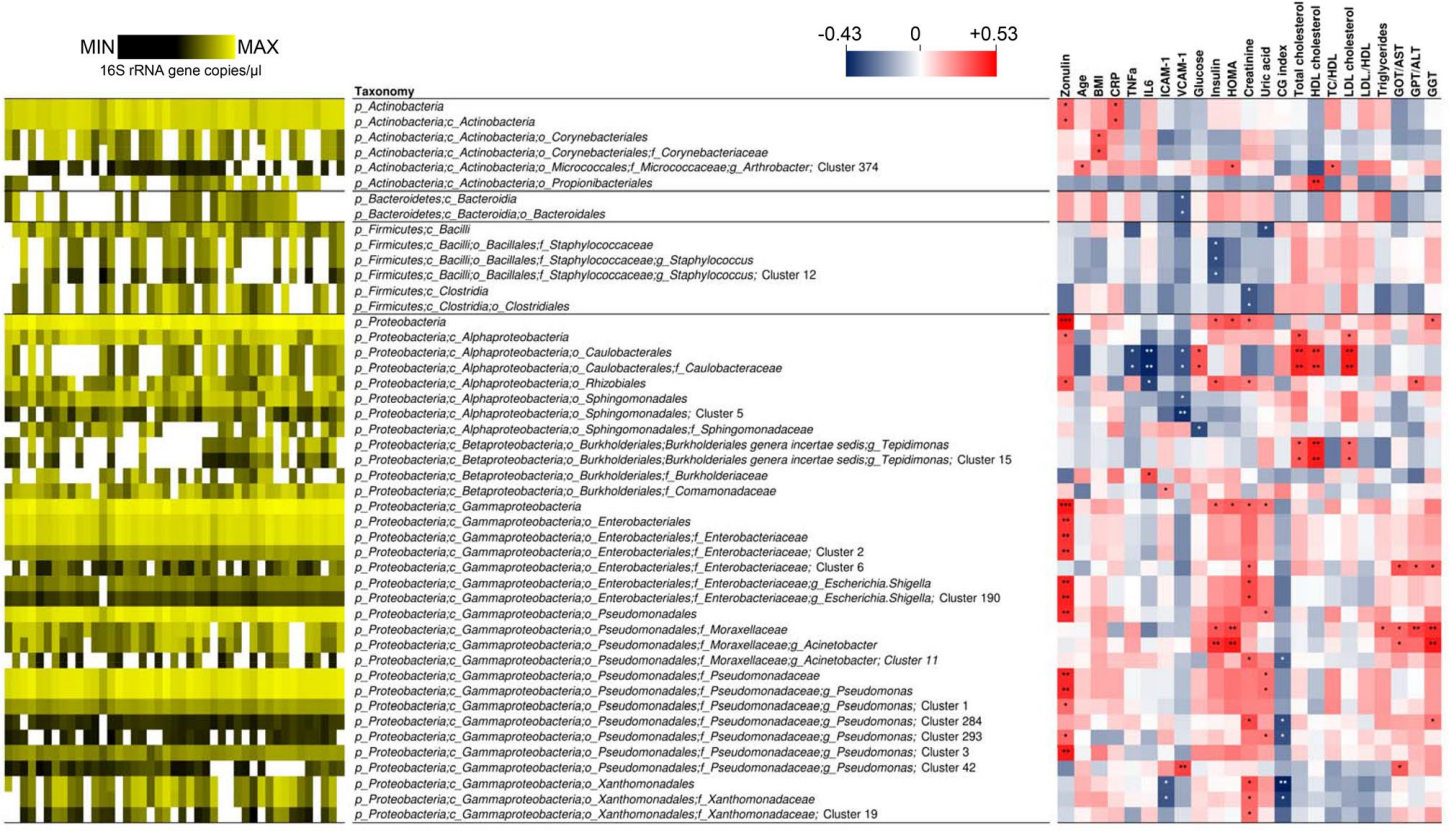
Correlations of the taxonomic units detected in blood (expressed as abundances normalized to 16S rRNA gene copies) toward age, BMI, and metabolic and functional markers determined in blood of the older subjects under study (n = 43). This figure only includes taxa whose abundance significantly correlated with at least one parameter. The heatmap on the left (black-yellow color gradient) shows the distribution among samples of the abundances of taxonomic units. White boxes in the black-yellow heatmap indicate that taxa that have not been detected in a specific sample. The heatmap on the right (blue-white-red color gradient) represents the Spearman’s correlation coefficient, ρ. Asterisks indicate the *P* value of the Kendall’s rank correlation: *P < 0.05; **P < 0.01; ***P < 0.001.

We performed correlation analyses between the same host markers described above and the taxonomic composition of blood bacterial DNA expressed as abundances normalized to 16S rRNA gene copies. Forty-seven taxa significantly correlated with at least one of the parameters considered (Fig. 5), about three quarters of which (33 taxa) belonging to the phylum *Proteobacteria*. The most numerous correlations with bacterial taxa were found for zonulin. In specific, seventeen bacterial taxa showed a significant positive correlation with zonulin levels, whereas none was inversely correlated. Zonulin levels mostly correlated with taxa within the class *γ-Proteobacteria*, i.e. the families *Enterobacteriaceae* and *Pseudomonadaceae*, and the genera *Pseudomonas* and *Escherichia/Shigella* (Fig. 5). In addition, a positive correlation with zonulin was observed for the phylum *Actinobacteria* and the class *Actinobacteria*, and for the class *γ-Proteobacteria* and the order Rhizobiales. The correlation between zonulin levels and the abundance of the phyla *Actinobacteria* and *Proteobacteria*, the order *γ-Proteobacteria*, the family *Pseudomonadaceae* and the genus *Pseudomonas* was also confirmed by the analysis of data generated by 16S rRNA gene profiling of the second set of blood samples collected from the older volunteers (n = 42) (Fig. S5).

Potential correlations were also investigated using taxonomic profiling data expressed as relative abundances (i.e., without normalization to 16S rRNA gene copies). This analysis revealed a lower number of significant correlations (Fig. S6). In particular, only one taxonomic unit, i.e. Cluster 3 (ascribed to the genus *Pseudomonas*), was directly correlated with zonulin levels, as also observed when the normalized taxonomic abundances were used. In addition, correlation analysis with taxonomic relative abundances confirmed the positive association of *Proteobacteria* with glycemia-related parameters and the inverse correlation of a few taxonomic units belonging to the *Pseudomonadaceae* family with the GC index (Fig. S6).

Overall, these results indicate that serum zonulin levels were mostly associated with blood bacterial DNA ascribed to the class *γ-Proteobacteria* and to the genus *Pseudomonas*.

## Discussion

The main objective of this study was to quantify and characterize the bacterial DNA present in the bloodstream of older individuals, and to identify possible significant correlations with different markers relevant for health status in older people.

Our results demonstrate that quantifiable and highly variable amounts of bacterial DNA is present in the venous blood of older subjects. Reports of other studies that were concerned with healthy adult volunteers have also provided evidence of bacterial DNA in human blood, suggesting that it is a common phenomenon even in apparently healthy subjects^3,9^. Nonetheless, our study is the first to report this observation in an older population.

Based on the limited literature data available, the amount of bacterial DNA in blood appears to be highly variable between individual subjects when assessed by quantification of 16S rRNA gene copy number^3^. For instance, the same protocol adopted in our study was previously used to quantify bacterial DNA in whole blood or buffy coat samples collected from healthy and obese subjects, and blood DNA concentrations ranging from zero up to about 8 × 10^7^ 16S rRNA gene copies per ml were obtained^3,21^. In our study, we found an average of 5.8 (± 3.1) × 10^6^ copies of 16S rRNA gene per ml of whole blood (minimum of 1.3 × 10^6^ and maximum of 1.2 × 10^7^); therefore, the older volunteers studied here harbor levels of circulating bacterial DNA not dissimilar from those reported for other groups of individuals^21^. Nonetheless, the data available to date are still too limited to draw conclusions.

The amount of bacterial DNA detected in blood was found to be significantly correlated with the serum level of zonulin. This significant correlation was confirmed when the analyses were repeated on a second set of blood samples, collected from the same older subjects after about 4 months, suggesting a stable association between these two variables in the group of subjects under investigation. To the best of our knowledge, the potential association between circulating bacterial DNA and zonulin has never been reported before. Zonulin (haptoglobin 2 precursor) is a physiologic modulator of intercellular tight junctions, which is involved in chronic inflammatory conditions^30^. In fact, zonulin has been shown to be increased in several pathologic states, including nonalcoholic fatty liver disease, obesity-associated insulin resistance, diabetes and sepsis^31^. Zonulin was also observed to be elevated in healthy aging^32^, supporting the hypothesis (recently verified in mouse model^17^), that intestinal permeability increases with age, promoting the progressive entry into the bloodstream of microbial factors which trigger the immune system leading to the low-grade systemic inflammation typically associated with aging. Therefore, inflammaging may promote chronic-degenerative diseases^16^. This mechanistic explanation for the onset of age-associated diseases is in agreement with the significant direct correlation observed between zonulin and the abundance of blood bacterial DNA in our study. However, the putative causal relationship between zonulin-mediated epithelial/endothelial permeability and bacterial DNAemia may be bidirectional because, whereas on one hand an increase in zonulin could lead to an increase in permeability and therefore to a greater translocation of bacterial DNA into the blood, on the other hand, the greater bacterial translocation in the blood might increase the local and systemic inflammation, which has been shown to induce zonulin expression^33^. Nonetheless, in the specific context of our study, it is relevant to consider that we did not report here the quantification of bacterial cells (either intact or broken), but we measured the abundance of a bacterial chromosomal region (the 16S rRNA gene), whose quantitative link with the presence of bacterial cells or their fragments cannot be extrapolated.

In the second part of this study, we taxonomically characterized the 16S rRNA genes detected in blood samples. Most of the bacterial DNA detected in blood in our study was ascribed to the phylum *Proteobacteria* and *Actinobacteria*, whereas *Firmicutes* and *Bacteroidetes* were less represented and not detected in all samples. A similar distribution of phyla was described by Paisse et al., who analyzed buffy coat, plasma and red blood cells in a cohort of thirty healthy blood donors^3^, and by Lelouvier et al., who studied a cohort of thirty-seven obese people^21^. The dominance of *Proteobacteria* and, secondarily, *Actinobacteria* was also reported by other independent studies, in which the authors investigated groups of individuals with different health conditions such as acute pancreatitis and cardiovascular disease, as well as healthy controls^9,34,35^. A high proportion of *Proteobacteria* was also detected in the DNA isolated from murine muscle, liver and brain tissues^23^, whereas in contrast, *Bacteroidetes* were reported to dominate the bacterial DNA detected in the blood of cats^36^, suggesting that the characteristics of DNAemia may be host-specific.

The third most abundant genus, detected in all but one of the samples, was the *Actinobacteria* taxon *Arthrobacter*. Interestingly, members of this genus have been frequently isolated as viable cells from wounds and blood (see Table 1 in^37^ which reported about 20 different *Arthrobacter* strains isolated from human blood and wound samples). Nonetheless, it must be also considered that the genus *Arthrobacter* has been reported in the list of laboratory contaminants detected in sequenced negative (blank) controls^38,39^.

It is generally assumed that bacterial translocation in blood principally depends on translocation from the intestine^21^; nonetheless, also the oral microbiota was suggested as a primary source of bacterial DNA in blood^40,41^. Interestingly, in the study by Vientos-Plotts on cats, the taxonomic composition of blood was found to resemble the lung microbiota, which was determined analyzing bronchoalveolar lavage fluids^36^. In our study, some of the most represented bacteria found in blood samples belong to taxa that have been reported as dominant members of the lung microbiota, such as *Pseudomonas*, *Sphyngomonadales* and *Acinetobacter*^42–44^. In addition, among the most represented taxa detected in blood, we also found members of the families *Comamonadaceae*, *Sphingomonadaceae*, and *Oxalobacteraceae*, which have been correlated with bronchial hypersensitivity in asthmatic patients^45^. These observations suggest that lung microbiota may be an additional source of bacterial DNA in blood. This hypothesis is not in contrast with our finding of a correlation between zonulin and blood bacterial DNA. In fact, zonulin, which is an emerging marker of intestinal permeability, was demonstrated to also be involved in the regulation of permeability in the lungs^46^. More generally, although the origin of the bacterial DNA detected in blood remains substantially unknown, the evident association with zonulin serum levels suggests that bacteria may have translocated into the blood through a para-cellular route at epithelial and/or endothelial cell surfaces, an event that plausibly occurred not only in the intestine.

The blood concentration of DNA ascribed to several bacterial taxa significantly correlated with a few host metabolic factors. In particular, we found a significant correlation of *γ-Proteobacteria* and *Pseudomonas* with serum zonulin for both sets of blood samples (i.e., blood collected at recruitment and after approximately four months). However, the actual physiological meaning of such associations (if any) cannot be easily predicted and, more importantly, we believe that the taxonomic results reported here must be interpreted with caution for the reasons discussed in Supplementary Discussion (Additional file 1).

## Conclusions

Based on the data reported here and in the context of previous reports of DNAemia in younger adults, the authors conclude that older individuals harbor detectable amounts of bacterial DNA in their blood and that it has a somewhat distinct taxonomic origin. In addition, our observed correlation between the concentration of bacterial DNA in blood and the serum levels of zonulin, an emerging marker of intestinal permeability, suggest that DNAemia is directly related with paracellular permeability, including but not necessarily limited to the epithelium of the gastrointestinal tract. We speculate that the bacterial DNA detected in blood, irrespective of the origin (gut, oral cavity or lung) and the form (free DNA, free bacterial cells, or bacteria internalized in blood cells), is indicative of a bacterial DNAemia that is not-silent, but may influence several aspects of host physiology. In particular, we propose that bacterial DNAemia may represent an important candidate biomarker helpful for the prognosis and/or prediction of metabolic and clinical pathologic conditions in the older subjects and can also be actively implicated in the development of inflammaging and aging associated diseases.

## Supplementary information

Supplementary Information 1.

Supplementary Information 2.

## Data Availability

The MaPLE trial was registered at the *International Standard Randomised Controlled Trial Number* (ISRCTN) registry under the code ISRCTN10214981. According to the data management plan of the MaPLE project, the datasets analyzed during the current study are available in the Dataverse repository, https://dataverse.unimi.it/dataverse/DNAemia-Zonulin (dataset https://doi.org/10.13130/RD_UNIMI/KGCL3D). Blood microbiomics sequencing reads have been also deposited in the European Nucleotide Archive (ENA) of the European Bioinformatics Institute under accession code PRJEB30560.
