# Supplementary material for: Bacterial DNAemia is associated with serum zonulin levels in older subjects
Source: Sci Rep. 2021 May 26;11:11054. doi: 10.1038/s41598-021-90476-0 (PMC8154904; doi:10.1038/s41598-021-90476-0)
Supplement: Supplementary file 1 — Supplementary Information 1. [file 41598_2021_90476_MOESM1_ESM.pdf]

# Bacterial DNAemia is associated with serum zonulin levels in older subjects

Giorgio Gargari[1], Giacomo Mantegazza[1], Valentina Taverniti[1], Cristian Del Bo'[2], Stefano Bernardi[2], Cristina Andres-Lacueva[3], Raul González-Domínguez[3], Paul A. Kroon[4], Mark S. Winterbone[4], Antonio Cherubini[5], Patrizia Riso[2], Simone Guglielmetti[1]*

[1]Division of Food Microbiology and Bioprocesses and [2]Division of Human Nutrition, Department of Food, Environmental and Nutritional Sciences (DeFENS), Università degli Studi di Milano, Milan, Italy

[3]Biomarkers and Nutrimetabolomics Laboratory, Department of Nutrition, Food Sciences and Gastronomy, University of Barcelona, Barcelona, Spain. CIBER de Fragilidad y Envejecimiento Saludable (CIBERFES), Instituto de Salud Carlos III, Barcelona, Spain.

[4]Quadram Institute Bioscience, Norwich Research Park, Norwich, UK;

[5]Geriatria, Accettazione geriatrica e Centro di ricerca per l'invecchiamento. IRCCS INRCA, Ancona, Italy

*Corresponding author. Mailing address: Via Celoria 2, 20133, Milano, Italia. E-mail: simone.guglielmetti@unimi.it. Tel: +39 02 503 19136. Fax: +39 02 503 19191

## Supplementary Table

**Table S1**. Characteristics of older subjects involved in the study (n=43). All data are presented as median and interquartile range (IQR) and as mean ± standard deviation (SD). BMI, body mass index; SBP, systolic blood pressure; DBP, diastolic blood pressure; TC, Total cholesterol, HDL-C, high density lipoprotein-cholesterol; LDL-C, low density lipoprotein-cholesterol; AST, aspartate aminotransferase; ALT, alanine aminotransferase; GGT, gamma-glutamyl transpeptidase; HOMA index, homeostasis model assessment index; C-G index, Cockcroft-Gault, sVCAM-1, vascular cells adhesion molecules-1; ICAM-1, intercellular cells adhesion molecules-1; CRP, C-reactive protein; TNF-α, tumour necrosis factor-alpha; IL-6, interleukin-6.

| **Variables** | **Median (IQR)** | **Mean (SD)** |
|---|---|---|
| 16S rRNA g.c./µl | 5444 (3522;7405) | 5776 ± 3110 |
| Zonulin (ng/ml) | 40.0 (34.4;48.6) | 41.1 ± 11.1 |
| Age (y) | 79.0 (72.0;87.5) | 79.2 ± 9.8 |
| BMI (kg/m2) | 25.4 (22.1;30.4) | 26.5 ± 5.8 |
| CRP (mg/l) | 5.0 (1.6;10.0) | 7.2 ± 7.7 |
| TNF-α (pg/ml) | 1.2 (1.0;1.8) | 1.7 ± 1.2 |
| IL-6 (pg/ml) | 3.7 (2.1;6.0) | 4.8 ± 4.3 |
| sICAM-1 (ng/ml) | 50.5 (41.4;67.2) | 55.9 ± 22.1 |
| sVCAM-1 (ng/ml) | 1017 (669;1356) | 1325 ± 1818 |
| Glucose (mg/dl) | 95.0 (85.0;115.0) | 115.2 ± 72.8 |
| Insuline (µU/ml) | 6.40 (5.05;8.95) | 8.63 ± 6.79 |
| HOMA index | 1.60 (1.15;2.41) | 3.02 ± 5.87 |
| Creatinine (mg/dl) | 0.81 (0.61;1.04) | 0.84 ± 0.31 |
| Uric Acid (mg/dl) | 5.00 (4.10;6.60) | 5.46 ± 1.86 |
| C-G index | 69.3 (53.4;82.9) | 75.1 ± 42.9 |
| TC (mg/dl) | 194 (170;239) | 197 ± 51 |
| HDL-C (mg/dl) | 46.0 (37.5;57.5) | 47.6 ± 15.7 |
| TC/HDL-C (ratio) | 4.0 (3.5;5.3) | 4.4 ± 1.2 |
| LDL-C (mg/dl) | 123.0 (94.5;146.0) | 121.5 ± 37.3 |
| LDL/HDL-C (ratio) | 2.53 (2.08;3.39) | 2.7 ± 0.78 |
| Triglycerides (mg/dl) | 113.0 (89.0;154.5) | 142.9 ± 97.4 |
| AST (U/l) | 17.0 (13.0;21.5) | 17.6 ± 6.1 |
| ALT (U/l) | 11.0 (8.0;17.0) | 13.2 ± 7.5 |
| GGT (U/l) | 21.0 (16.5;38.0) | 35.5 ± 38.9 |

## Supplementary Figures

**Fig. S1**. Metataxonomic analysis of data generated by 16S rRNA gene profiling for the first set of blood samples. **A**, rarefaction curves based on the richness of operative taxonomic units (OTUs). **B**, α-diversity analysis based on five different indexes, shown in order of increasing evenness weight in the algorithm, from observed species and Chao1 (no evenness considered) to InvSimpson index. **C**, principal coordinates analysis of generalized UniFrac distance (alpha=0.4); the first two coordinates are displayed; the percentage of variance explained is indicated in brackets. **D**, relative abundance of the most represented families and genera. Alpha diversity scores and UniFrac distance between samples were determined using Phyloseq R package [1].

**A**

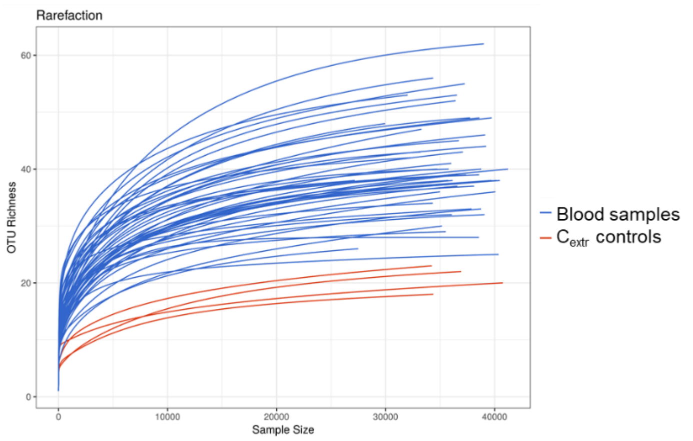


**B**

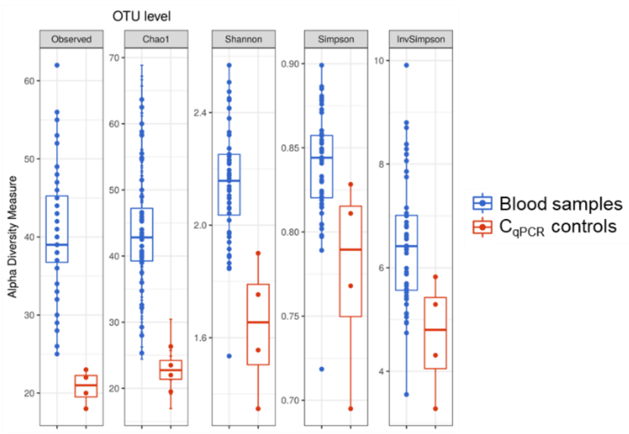


**C**

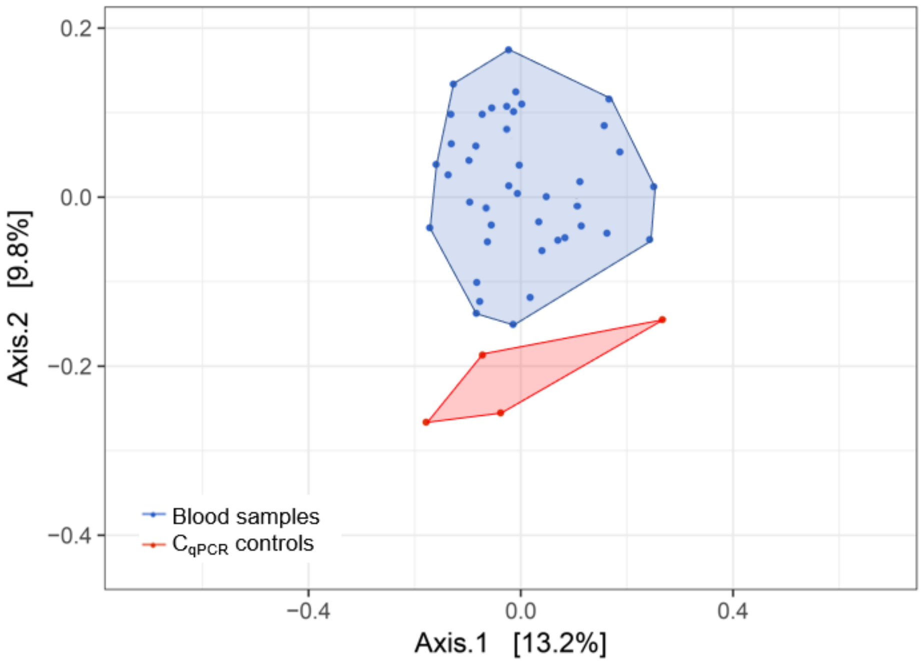


**D**

**Families**

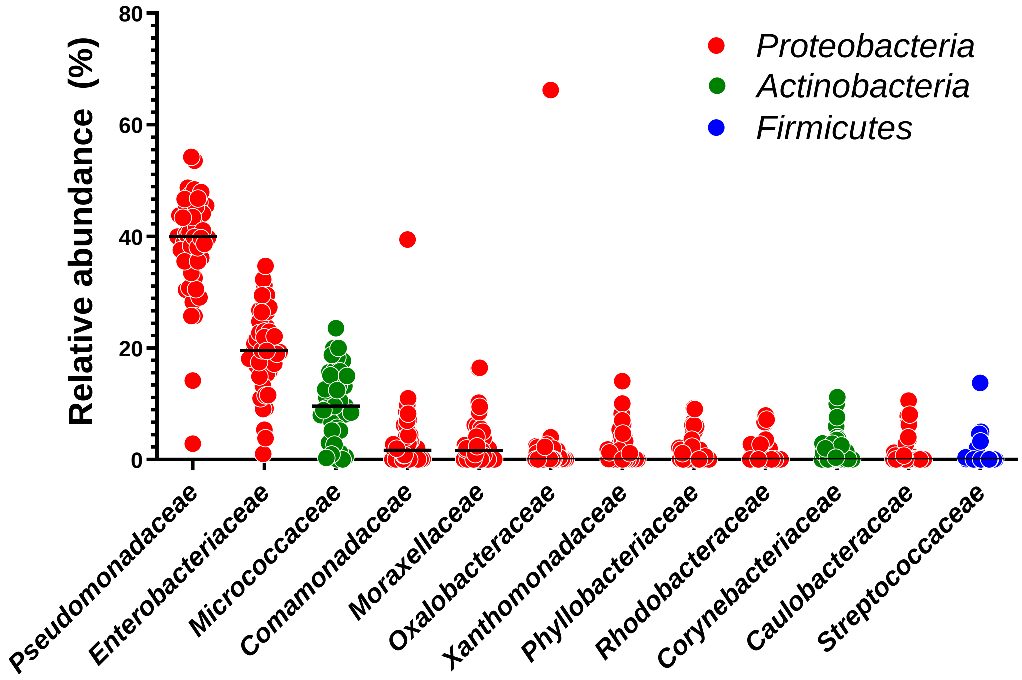


**Genera**

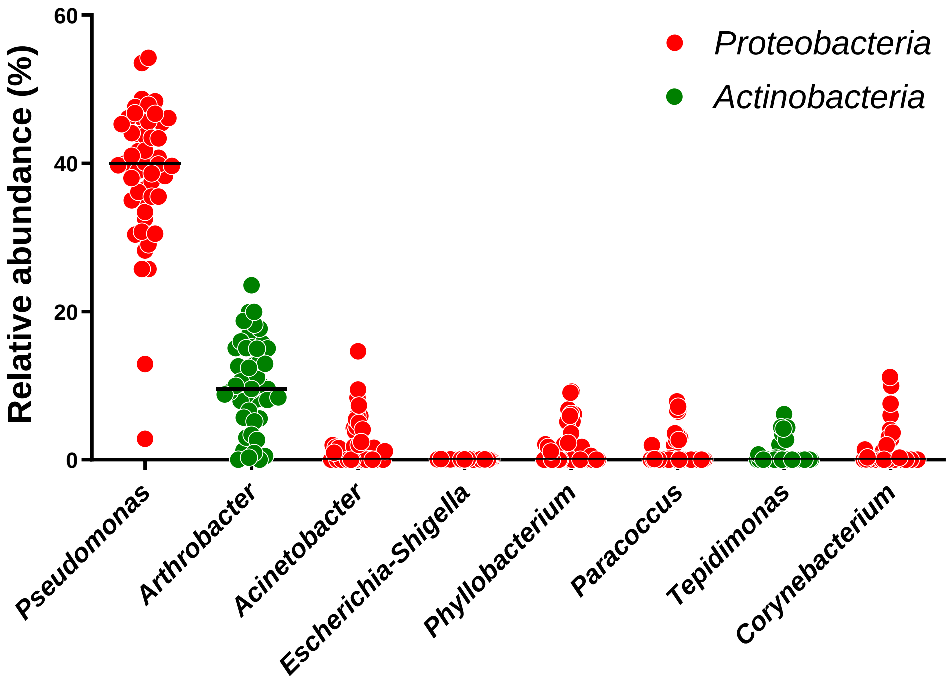

**Fig. S2**. Abundance in blood samples (B.S.) and controls ($C_{qPCR}$) of the main families (panels **A** and **C**) and genera (panels **B** and **D**) detected in blood (taxa are according to Fig. 4). Panels **A** and **B**, normalized abundances obtained multiplying the total 16S rRNA gene copies/µl by the percentage of each taxon in a specific sample. Panels **C** and **D**, relative abundances shown as percentage.

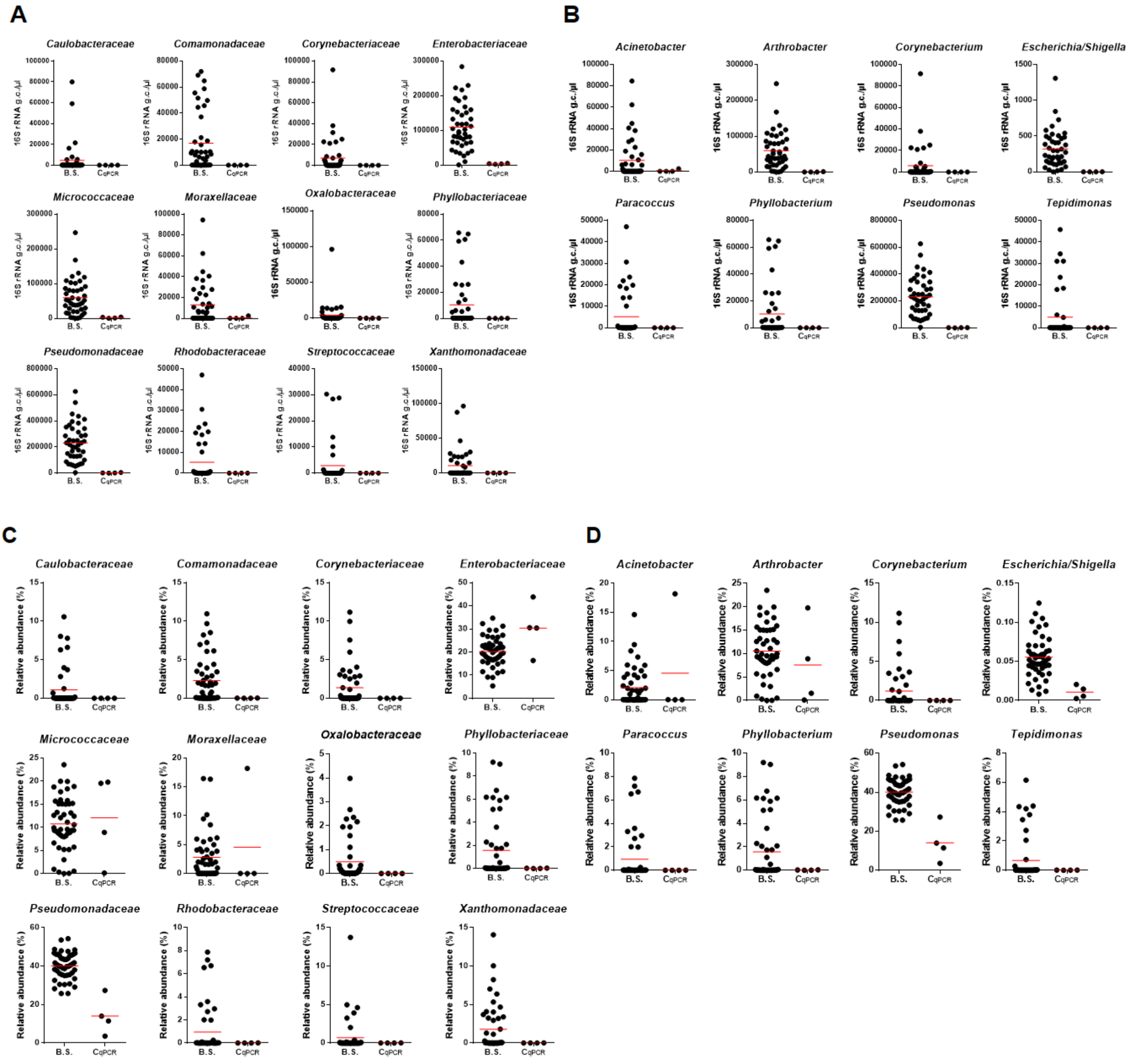

**Fig. S3**. Bacterial taxonomic profiling by 16S rRNA gene sequencing of additional controls and a blood sample (B.S.). $C_{extr}$, DNA extracted from ultrapure water; $C_{dEDTA}$, DNA extracted from commercial PBS; $C_{vEDTA}$, DNA extracted from commercial PBS passed through the vacutainer system. DNA extraction from all controls have been carried out following the same protocol used for the extraction of DNA from blood and was performed simultaneously to blood sample B.S. **A**, concentration of 16S rRNA gene copies (16S rRNA g.c./µl) determined by qPCR. **B**, number of sequencing reads assigned to operational taxonomic units (OTUs) per sample. **C**, taxonomic composition of each sample at OTU level; each Cluster corresponds to an OTU; genera of main Clusters detected in B.S. are specified on the right. **D**, distribution of taxonomic Clusters (i.e., OTUs) detected in the analyzed samples; the bacterial genus to which each cluster belong is shown on the right; white cells in the heatmap indicate that the bacterial taxon was not detected in a specific sample.

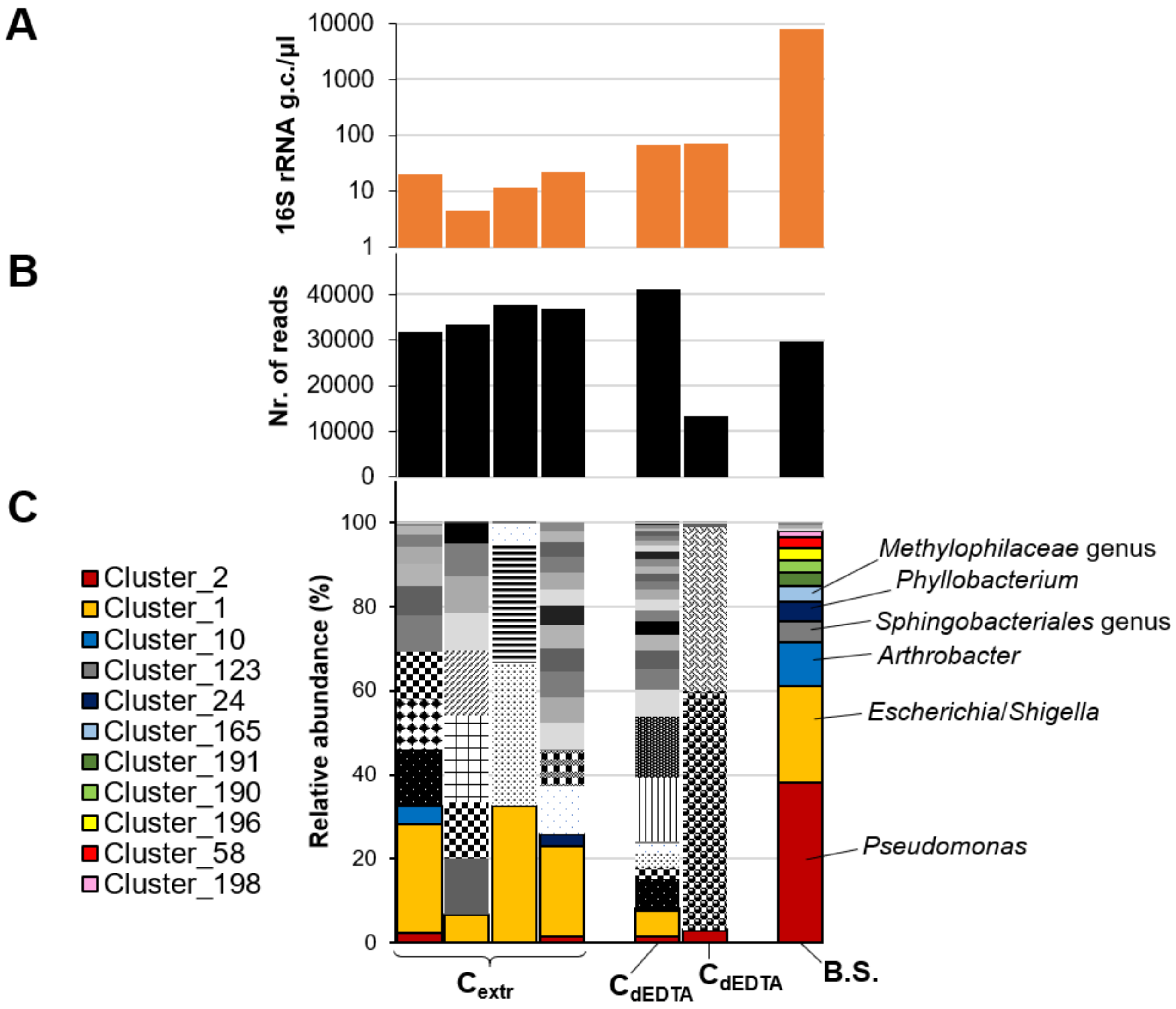

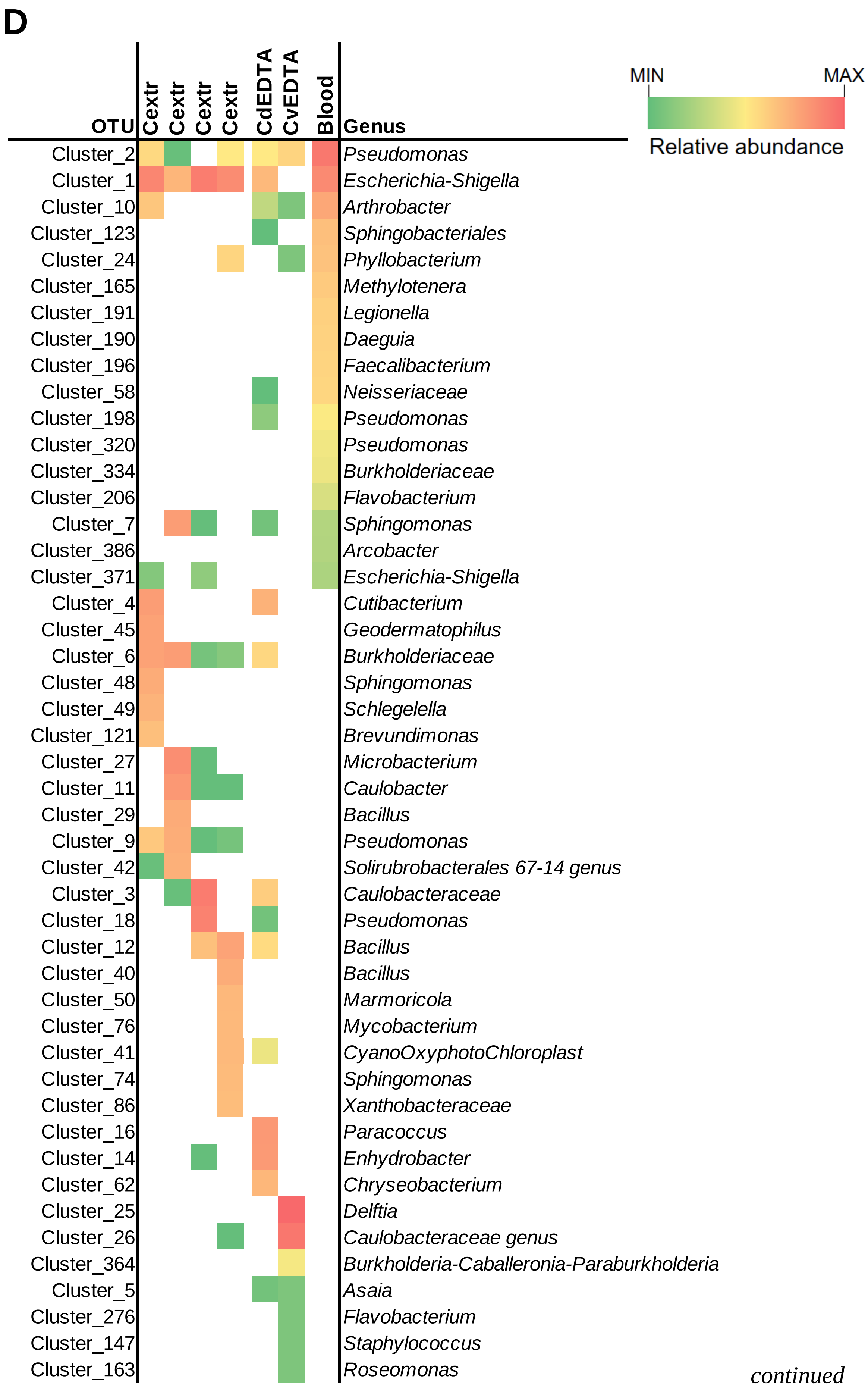
D
OTU
Cextr
Cextr
Cextr
Cextr
CdEDTA
CvEDTA
Blood
Genus
MIN
MAX
Relative abundance
Cluster_2 Pseudomonas
Cluster_1 Escherichia-Shigella
Cluster_10 Arthrobacter
Cluster_123 Sphingobacteriales
Cluster_24 Phyllobacterium
Cluster_165 Methylotenera
Cluster_191 Legionella
Cluster_190 Daeguia
Cluster_196 Faecalibacterium
Cluster_58 Neisseriaceae
Cluster_198 Pseudomonas
Cluster_320 Pseudomonas
Cluster_334 Burkholderiaceae
Cluster_206 Flavobacterium
Cluster_7 Sphingomonas
Cluster_386 Arcobacter
Cluster_371 Escherichia-Shigella
Cluster_4 Cutibacterium
Cluster_45 Geodermatophilus
Cluster_6 Burkholderiaceae
Cluster_48 Sphingomonas
Cluster_49 Schlegelella
Cluster_121 Brevundimonas
Cluster_27 Microbacterium
Cluster_11 Caulobacter
Cluster_29 Bacillus
Cluster_9 Pseudomonas
Cluster_42 Solirubrobacterales 67-14 genus
Cluster_3 Caulobacteraceae
Cluster_18 Pseudomonas
Cluster_12 Bacillus
Cluster_40 Bacillus
Cluster_50 Marmoricola
Cluster_76 Mycobacterium
Cluster_41 CyanoOxyphotoChloroplast
Cluster_74 Sphingomonas
Cluster_86 Xanthobacteraceae
Cluster_16 Paracoccus
Cluster_14 Enhydrobacter
Cluster_62 Chryseobacterium
Cluster_25 Delftia
Cluster_26 Caulobacteraceae genus
Cluster_364 Burkholderia-Caballeronia-Paraburkholderia
Cluster_5 Asaia
Cluster_276 Flavobacterium
Cluster_147 Staphylococcus
Cluster_163 Roseomonas

*continued*

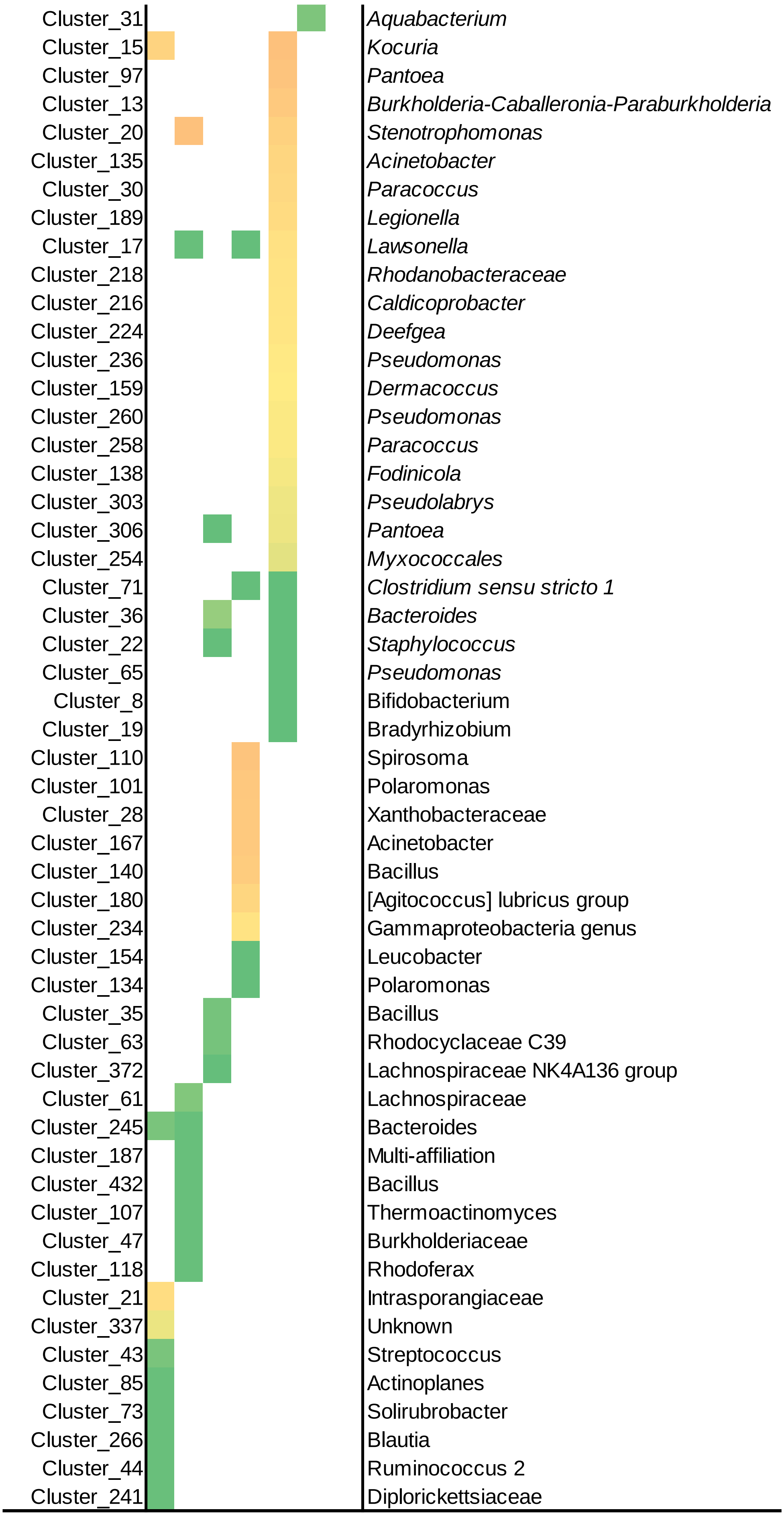
Cluster_31 Aquabacterium
Cluster_15 Kocuria
Cluster_97 Pantoea
Cluster_13 Burkholderia-Caballeronia-Paraburkholderia
Cluster_20 Stenotrophomonas
Cluster_135 Acinetobacter
Cluster_30 Paracoccus
Cluster_189 Legionella
Cluster_17 Lawsonella
Cluster_218 Rhodanobacteraceae
Cluster_216 Caldicoprobacter
Cluster_224 Deefgea
Cluster_236 Pseudomonas
Cluster_159 Dermacoccus
Cluster_260 Pseudomonas
Cluster_258 Paracoccus
Cluster_138 Fodinicola
Cluster_303 Pseudolabrys
Cluster_306 Pantoea
Cluster_254 Myxococcales
Cluster_71 Clostridium sensu stricto 1
Cluster_36 Bacteroides
Cluster_22 Staphylococcus
Cluster_65 Pseudomonas
Cluster_8 Bifidobacterium
Cluster_19 Bradyrhizobium
Cluster_110 Spirosoma
Cluster_101 Polaromonas
Cluster_28 Xanthobacteraceae
Cluster_167 Acinetobacter
Cluster_140 Bacillus
Cluster_180 [Agitococcus] lubricus group
Cluster_234 Gammaproteobacteria genus
Cluster_154 Leucobacter
Cluster_134 Polaromonas
Cluster_35 Bacillus
Cluster_63 Rhodocyclaceae C39
Cluster_372 Lachnospiraceae NK4A136 group
Cluster_61 Lachnospiraceae
Cluster_245 Bacteroides
Cluster_187 Multi-affiliation
Cluster_432 Bacillus
Cluster_107 Thermoactinomyces
Cluster_47 Burkholderiaceae
Cluster_118 Rhodoferax
Cluster_21 Intrasporangiaceae
Cluster_337 Unknown
Cluster_43 Streptococcus
Cluster_85 Actinoplanes
Cluster_73 Solirubrobacter
Cluster_266 Blautia
Cluster_44 Ruminococcus 2
Cluster_241 Diplorickettsiaceae

**Fig. S4**. Results of the qPCR experiments performed to assess the “carrier effect” during DNA extraction from EDTA tube. The same volume of milliQ water and λ phage dilutions were added to EDTA tubes. - , DNA extracted from EDTA tube containing MRD solution; mQ, EDTA tube containing MRD solution with the addition of the same volume of Milli-Q water used to prepare the λ DNA dilutions.  Cq, cycle threshold used for qPCR quantification. DEB, DNA elution buffer of the commercial kit used for DNA extraction.

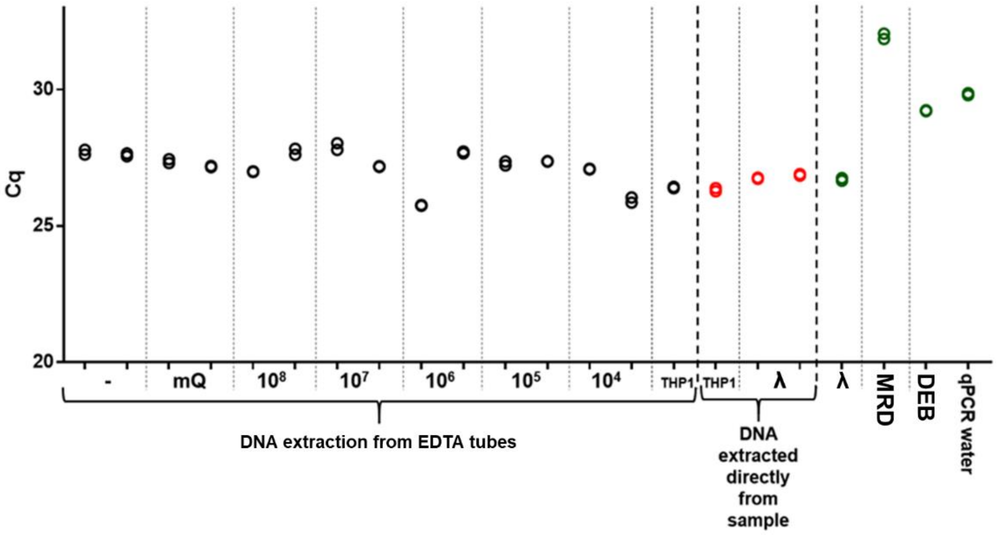


| | Sample | Cq |
|---|---|---|
| DNA extraction from EDTA tubes | - | 27.31 |
| | - | 27.45 |
| | - | 27.16 |
| | - | 27.2 |
| | mQ | 27.79 |
| | mQ | 27.62 |
| | mQ | 27.56 |
| | mQ | 27.66 |
| | λ 10^8 copies | 26.98 |
| | λ 10^8 copies | 27.00 |
| | λ 10^8 copies | 27.61 |
| | λ 10^8 copies | 27.83 |
| | λ 10^7 copies | 28.03 |
| | λ 10^7 copies | 27.79 |
| | λ 10^7 copies | 27.17 |
| | λ 10^7 copies | 27.19 |
| | λ 10^6 copies | 25.74 |
| | λ 10^6 copies | 25.77 |
| | λ 10^6 copies | 27.73 |
| | λ 10^6 copies | 27.67 |
| | λ 10^5 copies | 27.36 |
| | λ 10^5 copies | 27.22 |
| | λ 10^5 copies | 27.36 |
| | λ 10^5 copies | 27.38 |
| | λ 10^4 copies | 27.1 |
| | λ 10^4 copies | 27.07 |
| | λ 10^4 copies | 25.86 |
| | λ 10^4 copies | 26.04 |
| | THP-1 DNA | 26.27 |
| | THP-1 DNA | 26.39 |

| | Sample | Cq |
|---|---|---|
| DNA extracted directly from sample | THP-1 DNA | 26.39 |
| | THP-1 DNA | 26.43 |
| | λ DNA solution | 26.73 |
| | λ DNA solution | 26.77 |
| | λ DNA solution | 26.9 |
| | λ DNA solution | 26.85 |
| Template in qPCR | λ 2.9 x 10^8 copies | 26.66 |
| | λ 2.9 x 10^8 copies | 26.75 |
| | MRD solution | 32.05 |
| | MRD solution | 31.86 |
| | DNA elution buffer | 29.21 |
| | DNA elution buffer | 29.24 |
| | qPCR water | 29.8 |
| | qPCR water | 29.87 |

**Fig. S5**. Abundance of 16S rRNA gene copies of taxonomic units detected in second set of blood samples (n=42) that significantly correlated with the serum levels of zonulin. ρ, Spearman's rank correlation coefficient; *P*, P value of the Kendall's rank correlation. Taxa that resulted significantly correlated with zonulin also from the analysis of the first set of blood samples are indicated in bold and red color.

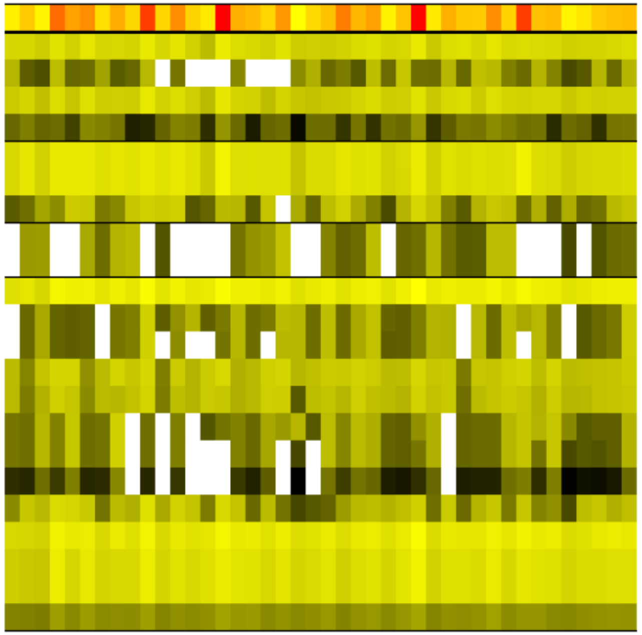

| | Zonulin ρ | Zonulin *P* |
|---|---|---|
| *16S rRNA gene copies* | 0,49 | ** |
| ***p_Actinobacteria*** | 0,45 | ** |
| *p_Actinobacteria;c_Actinobacteria;o_Frankiales* | 0,41 | ** |
| *p_Actinobacteria;c_Actinobacteria;o_Micrococcales* | 0,39 | ** |
| *p_Actinobacteria;c_Actinobacteria;o_Micrococcales;f_Microbacteriaceae; Candidatus Aquiluna;* Cluster 7 | 0,27 | * |
| *p_Bacteroidetes* | 0,34 | * |
| *p_Bacteroidetes;c_Bacteroidia* | 0,34 | * |
| *p_Bacteroidetes;c_Sphingobacteriia;o_Sphingobacteriales* | 0,41 | ** |
| *p_Patescibacteria;c_Saccharimonadia* | 0,32 | * |
| *p_Patescibacteria;c_Saccharimonadia;o_Saccharimonadales* | 0,32 | * |
| ***p_Proteobacteria*** | 0,38 | * |
| *p_Proteobacteria;c_Alphaproteobacteria;o_Caulobacterales* | 0,34 | * |
| *p_Proteobacteria;c_Alphaproteobacteria;o_Caulobacterales;f_Caulobacteraceae* | 0,42 | ** |
| *p_Proteobacteria;c_Betaproteobacteri* | 0,46 | ** |
| *p_Proteobacteria;c_Betaproteobacteria;o_Burkholderiales;f_Burkholderiaceae* | 0,42 | ** |
| *p_Proteobacteria;c_Betaproteobacteria;o_Rhodocyclales;f_Azonexaceae* | 0,45 | ** |
| *p_Proteobacteria;c_Betaproteobacteria;o_Rhodocyclales;f_Azonexaceae;g_Dechloromonas* | 0,44 | ** |
| *p_Proteobacteria;c_Betaproteobacteria;o_Rhodocyclales;f_Azonexaceae;g_Dechloromonas;* Cluster 9 | 0,36 | * |
| *p_Proteobacteria;c_Deltaproteobacteria* | 0,28 | * |
| ***p_Proteobacteria;c_Gammaproteobacteria*** | 0,41 | ** |
| ***p_Proteobacteria;c_Gammaproteobacteria;o_Pseudomonadales;f_Pseudomonadaceae*** | 0,29 | * |
| ***p_Proteobacteria;c_Gammaproteobacteria;o_Pseudomonadales;f_Pseudomonadaceae;g_Pseudomonas*** | 0,29 | * |
| ***p_Proteobacteria;c_Gammaproteobacteria;o_Pseudomonadales;f_Pseudomonadaceae;g_Pseudomonas;* Cluster 1** | 0,32 | * |

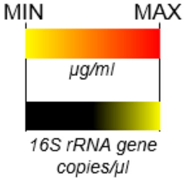


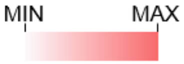

**Fig. S6**. Correlations of the taxonomic units detected in blood (expressed as relative abundances) toward age, BMI, and metabolic and functional markers determined in blood of the older subjects under study (n=43). This figure only includes taxa whose abundance significantly correlated with at least one parameter. The heatmap (blue-white-red color gradient) represents the Spearman's correlation coefficient, ρ. Asterisks indicate the P value of the Kendall's rank correlation: *, P<0.05; **, P<0.01.

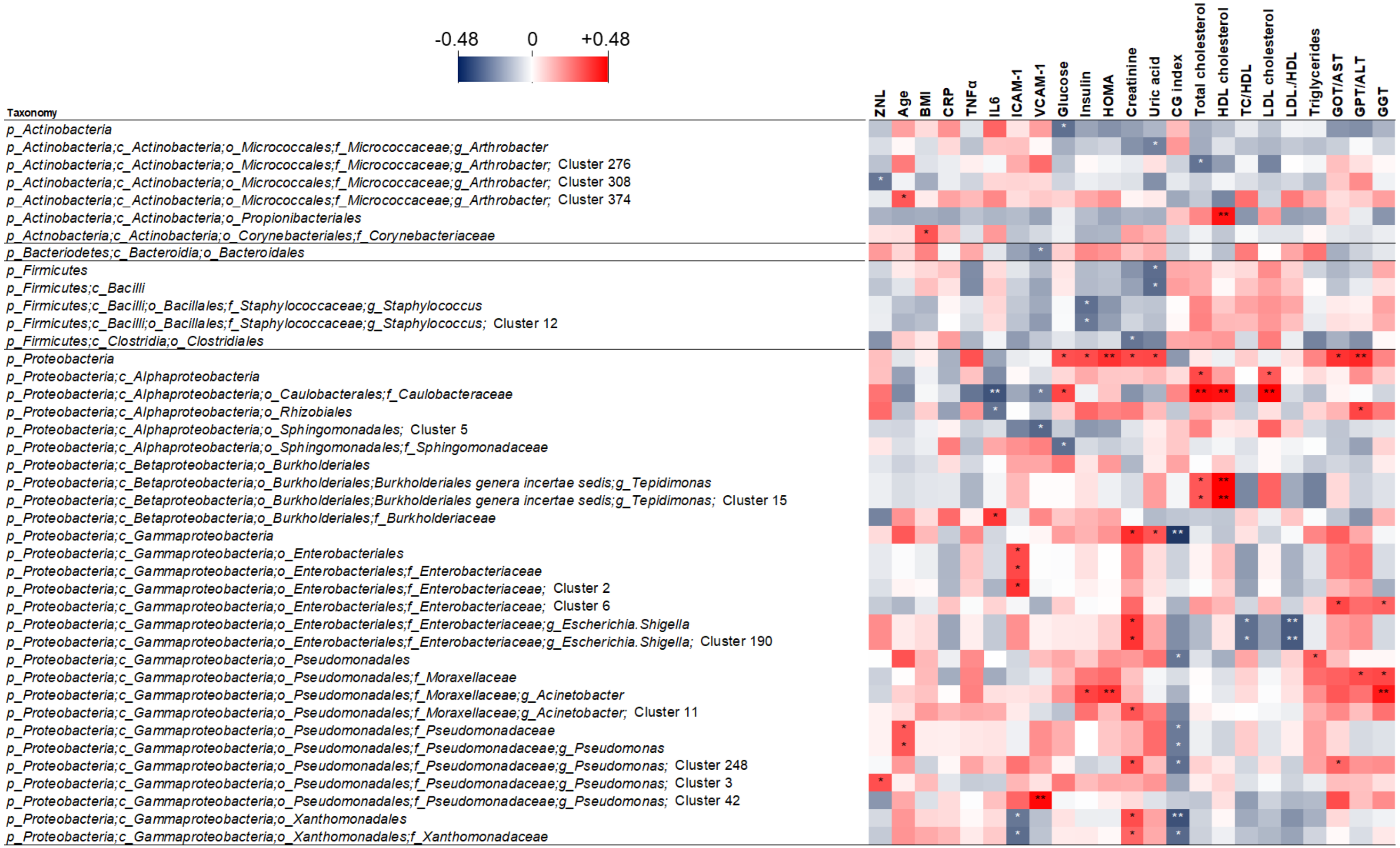

## Supplementary Discussion

### Technical issues concerning zonulin quantification

In this study, zonulin quantification in serum samples was carried out by means of the most commonly used commercial ELISA kit. Recently, the specificity of this and others ELISA assays has been questioned [2] and, consequently, it was suggested to interpret with caution data collected as direct assessment of intestinal permeability [3]. In this context, it is noteworthy that already Scheffer and colleagues [4], previously identified through the use of the same kit a variety of proteins structurally related to zonulin (in particular properdin). Consequently, the authors suggested that although the assay was not specific for pre-haptoglobin2 quantification, other members of permeability-regulating proteins belonging to the mannose-associated serine protease family could be determined [4].

### Technical issues concerning the detection and taxonomic profiling of bacteria DNA in blood

In a recent publication, circulating cell-free DNA isolated from human blood plasma was subjected to massive shotgun sequencing [5]; more than half of the identified contigs had little or no homology with sequences in available databases and, interestingly, were assigned to hundreds of entirely novel microbial taxa. In our study, we did not find such a large presence of unknown microorganisms. Nonetheless, two main aspects distinguish the research by Kowarsky et al. from ours: (i) we performed 16S rRNA gene profiling and not shotgun metagenomic sequencing and (ii) we analyzed DNA isolated from whole blood and not plasma. This second aspect is particularly important considering the presence of bacterial DNA in blood cells such as erythrocytes and antigen-presenting cells [6,7].

In this study, the bacterial DNA isolated from blood was taxonomically profiled through MiSeq sequencing of 16S rRNA gene amplicons. We presented above numerous similarities, both quantitatively (i.e., abundance of 16S rRNA gene copies) and qualitatively (i.e., detected taxa), between the results of our study ad what reported in several other studies available in literature. However, none of the papers we referenced above focused specifically on the evaluation of potential contaminant DNA, originating from any possible experimental step. The use of 16S rRNA gene profiling for the bacterial taxonomic characterization of low microbial biomass samples, such as blood, has been criticized as being at high risk of microbial contamination that may occur at any step of the protocol, from sample collection until sequencing [8,9]. In our study, we analyzed several control samples to assess the potential presence of contaminants in labware (e.g. vacutainer and EDTA tubes) and reagents (e.g. solutions used during extraction, library preparation, sequencing, and qPCR). According to qPCR experiments, we always detected in control samples a quantity of bacterial DNA much lower than that quantified in blood samples, suggesting the potential contaminants should not have significantly affected the taxonomic profiling of blood samples. However, the confirmation of a significant correlation between zonulin and 16S rRNA gene copies in blood (total and ascribed to *Pseudomonas*) also in the second set of blood samples investigated supports the conclusion that the bacterial DNA detected in blood largely do not derive from contamination. Nonetheless, it is also important to mention that most of the bacterial

genera detected in blood in our study have been reported as contaminants occurring during microbiome research in other studies (reviewed in [8]).

Considering the relative abundance of bacterial taxa detected in blood and control samples, we hypothesize that the most probable contaminants belong to the families *Enterobacteriaceae*, *Micrococcaceae* and *Moraxellaceae* (the second, third and fifth most abundant families detected in blood, respectively), whereas at least most part of the DNA ascribed to *Pseudomonadaceae* (the most abundant family detected in blood) is less likely to derive from contaminants. Lists of bacterial taxa that were identified in negative controls during different independent studies have been proposed [8,10], cataloging up to 70 different genera to be considered as potential contaminants [8]. These lists contain numerous *Proteobacteria* including *Pseudomonas*, which was found to be the most prevalent and abundant bacterial genus in the blood samples investigated in our study. *Pseudomonas* is a ubiquitous bacterium, which colonizes numerous environments, such as soil, water and various plant and animal organisms, due to minimal survival requirements and remarkable adaptation ability [11]. Notably, *Pseudomonas* is also one of the microorganisms most frequently isolated from patients with bacteremia, particularly the species *P. aeruginosa* [12]. In this report, the partial sequence of the 16S rRNA gene belonging to the most prevalent and abundant OTUs found in the analyzed blood samples (Cluster 1 and Cluster 3, Fig. 5) shared 100% similarity with *P. fluorescens* and other species of the same phylogenetic lineage. Although far less pathogenic than *P. aeruginosa*, *P. fluorescens* has been often reported as the aetiologic agent of opportunistic infections in lungs, mouth, stomach, urinary tract, skin, and, most commonly, blood [13,14]. Notably, *P. fluorescens* is recognized as the most important cause of iatrogenic sepsis, attributed to contaminated blood transfusion or contaminated equipment used in intravenous infusions [15-17]. Although the literature evidence discussed above suggests that *P. fluorescens* and related species can be contaminants (see Supplementary Discussion in Additional file 1), on the other hand, these bacteria were also reported to possess numerous functional properties that support their survival and growth in mammalian hosts [14]. Furthermore, an interesting association was found between the presence of serum antibodies against the I2 peptide encoded by *P. fluorescens* and Crohn's disease [18], celiac disease [19], ankylosing spondylitis [20], and chronic granulomatous disease [21]. In addition, *P. fluorescens* was reported to be regularly cultured from clinical samples even in the absence of acute infection [14]. Finally, *P. fluorescens* was demonstrated to induce zonulin expression and decreased intestinal permeability in a time dependent manner in an in vitro model of intestinal epithelium [22]. In the same study, the authors found increased zonulin levels and higher abundance of *Pseudomonas* 16S rRNA gene copies (as determined through qPCR with genus-specific primers) in coronary artery disease (CAD) patients compared to non-CAD subjects [22]. Altogether, these reports support the hypothesis that human-adapted *P. fluorescens* strains constitute low-abundance indigenous members of the microbial ecosystem of various body sites, such as the lungs, mouth, and stomach [14,23-25]. Contextually, we can speculate that certain *P. fluorescens*-related strains are highly adaptable and poorly pathogenic members of the microbiota in several body sites that may frequently translocate into the bloodstream, providing a dominant contribution to bacterial DNAemia. However, we are conscious that our results do not conclusively demonstrate the actual presence of *Pseudomonas* (cells or free DNA) in blood. We believe that DNA-

independent methods, for instance based on the use of electron microscopy or bacterium-specific antibodies, could contribute to the unambiguous demonstration of the presence of these bacteria in human blood.

## References


1 McMurdie, P. J. & Holmes, S. phyloseq: an R package for reproducible interactive analysis and graphics of microbiome census data. *PloS one* **8**, e61217, doi:10.1371/journal.pone.0061217 (2013).

2 Ajamian, M., Steer, D., Rosella, G. & Gibson, P. R. Serum zonulin as a marker of intestinal mucosal barrier function: May not be what it seems. *PloS one* **14**, e0210728, doi:10.1371/journal.pone.0210728 (2019).

3 Aydin, O. *et al.* Examination of plasma zonulin levels in bipolar I disorder: a case-control study with follow-up. *J Neural Transm (Vienna)* **127**, 1419-1426, doi:10.1007/s00702-020-02234-7 (2020).

4 Scheffler, L. *et al.* Widely Used Commercial ELISA Does Not Detect Precursor of Haptoglobin2, but Recognizes Properdin as a Potential Second Member of the Zonulin Family. *Front Endocrinol (Lausanne)* **9**, 22, doi:10.3389/fendo.2018.00022 (2018).

5 Kowarsky, M. *et al.* Numerous uncharacterized and highly divergent microbes which colonize humans are revealed by circulating cell-free DNA. *Proceedings of the National Academy of Sciences of the United States of America* **114**, 9623-9628, doi:10.1073/pnas.1707009114 (2017).

6 Potgieter, M., Bester, J., Kell, D. B. & Pretorius, E. The dormant blood microbiome in chronic, inflammatory diseases. *FEMS Microbiol Rev* **39**, 567-591, doi:10.1093/femsre/fuv013 (2015).

7 Minasyan, H. Mechanisms and pathways for the clearance of bacteria from blood circulation in health and disease. *Pathophysiology : the official journal of the International Society for Pathophysiology* **23**, 61-66, doi:10.1016/j.pathophys.2016.03.001 (2016).

8 Eisenhofer, R. *et al.* Contamination in Low Microbial Biomass Microbiome Studies: Issues and Recommendations. *Trends in microbiology* **27**, 105-117, doi:10.1016/j.tim.2018.11.003 (2019).

9 Weyrich, L. S. *et al.* Laboratory contamination over time during low-biomass sample analysis. *Molecular ecology resources* **19**, 982-996, doi:10.1111/1755-0998.13011 (2019).

10 Salter, S. J. *et al.* Reagent and laboratory contamination can critically impact sequence-based microbiome analyses. *BMC biology* **12**, 87, doi:10.1186/s12915-014-0087-z (2014).

11 Goldberg, J. B. Pseudomonas: global bacteria. *Trends in microbiology* **8**, 55-57, doi:10.1016/s0966-842x(99)01671-6 (2000).

12 Nielsen, S. L. The incidence and prognosis of patients with bacteremia. *Danish medical journal* **62** (2015).

13 Azam, M. W. & Khan, A. U. Updates on the pathogenicity status of Pseudomonas aeruginosa. *Drug discovery today* **24**, 350-359, doi:10.1016/j.drudis.2018.07.003 (2019).

14 Scales, B. S., Dickson, R. P., LiPuma, J. J. & Huffnagle, G. B. Microbiology, genomics, and clinical significance of the Pseudomonas fluorescens species complex, an unappreciated colonizer of humans. *Clinical microbiology reviews* **27**, 927-948, doi:10.1128/CMR.00044-14 (2014).

15 Benito, N. *et al.* Outbreak of Pseudomonas fluorescens bloodstream infection in a coronary care unit. *The Journal of hospital infection* **82**, 286-289, doi:10.1016/j.jhin.2012.09.008 (2012).

16 Centers for Disease, C. & Prevention. Update: Delayed onset Pseudomonas fluorescens bloodstream infections after exposure to contaminated heparin flush--Michigan and South Dakota, 2005-2006. *MMWR. Morbidity and mortality weekly report* **55**, 961-963 (2006).

17 Centers for Disease, C. & Prevention. Pseudomonas bloodstream infections associated with a heparin/saline flush--Missouri, New York, Texas, and Michigan, 2004-2005. *MMWR. Morbidity and mortality weekly report* **54**, 269-272 (2005).

18 Landers, C. J. *et al.* Selected loss of tolerance evidenced by Crohn's disease-associated immune responses to auto- and microbial antigens. *Gastroenterology* **123**, 689-699, doi:10.1053/gast.2002.35379 (2002).

19 Ashorn, S. *et al.* Elevated serum anti-Saccharomyces cerevisiae, anti-I2 and anti-OmpW antibody levels in patients with suspicion of celiac disease. *Journal of clinical immunology* **28**, 486-494, doi:10.1007/s10875-008-9200-9 (2008).

20 Mundwiler, M. L. *et al.* Inflammatory bowel disease serologies in ankylosing spondylitis patients: a pilot study. *Arthritis research & therapy* **11**, R177, doi:10.1186/ar2866 (2009).

21 Yu, J. E. *et al.* High levels of Crohn's disease-associated anti-microbial antibodies are present and independent of colitis in chronic granulomatous disease. *Clinical immunology* **138**, 14-22, doi:10.1016/j.clim.2010.08.003 (2011).

22 Li, C. *et al.* Zonulin Regulates Intestinal Permeability and Facilitates Enteric Bacteria Permeation in Coronary Artery Disease. *Sci Rep* **6**, 29142, doi:10.1038/srep29142 (2016).

23 Diaz, P. I. *et al.* Transplantation-associated long-term immunosuppression promotes oral colonization by potentially opportunistic pathogens without impacting other members of the salivary bacteriome. *Clinical and vaccine immunology : CVI* **20**, 920-930, doi:10.1128/CVI.00734-12 (2013).

24 Patel, S. K. *et al.* Pseudomonas fluorescens-like bacteria from the stomach: a microbiological and molecular study. *World journal of gastroenterology* **19**, 1056-1067, doi:10.3748/wjg.v19.i7.1056 (2013).

25 Dickson, R. P. *et al.* Changes in the lung microbiome following lung transplantation include the emergence of two distinct Pseudomonas species with distinct clinical associations. *PloS one* **9**, e97214, doi:10.1371/journal.pone.0097214 (2014).
